# Local Chromatin Features Including PU.1 and IKAROS Binding and H3K4 Methylation Shape the Repertoire of Immunoglobulin Kappa Genes Chosen for V(D)J Recombination

**DOI:** 10.3389/fimmu.2017.01550

**Published:** 2017-11-17

**Authors:** Louise S. Matheson, Daniel J. Bolland, Peter Chovanec, Felix Krueger, Simon Andrews, Hashem Koohy, Anne E. Corcoran

**Affiliations:** ^1^Nuclear Dynamics Programme, Babraham Institute, Cambridge, United Kingdom; ^2^Bioinformatics Group, Babraham Institute, Cambridge, United Kingdom

**Keywords:** V(D)J recombination, immunoglobulin kappa, epigenetic regulation, chromatin state, PU.1, IKAROS

## Abstract

V(D)J recombination is essential for the generation of diverse antigen receptor (AgR) repertoires. In B cells, immunoglobulin kappa (*Igκ*) light chain recombination follows immunoglobulin heavy chain (*Igh*) recombination. We recently developed the DNA-based VDJ-seq assay for the unbiased quantitation of *Igh* VH and DH repertoires. Integration of VDJ-seq data with genome-wide datasets revealed that two chromatin states at the recombination signal sequence (RSS) of VH genes are highly predictive of recombination in mouse pro-B cells. It is unknown whether local chromatin states contribute to Vκ gene choice during *Igκ* recombination. Here we adapt VDJ-seq to profile the *Igκ* VκJκ repertoire and present a comprehensive readout in mouse pre-B cells, revealing highly variable Vκ gene usage. Integration with genome-wide datasets for histone modifications, DNase hypersensitivity, transcription factor binding and germline transcription identified PU.1 binding at the RSS, which was unimportant for *Igh*, as highly predictive of whether a Vκ gene will recombine or not, suggesting that it plays a binary, all-or-nothing role, priming genes for recombination. Thereafter, the frequency with which these genes recombine was shaped both by the presence and level of enrichment of several other chromatin features, including H3K4 methylation and IKAROS binding. Moreover, in contrast to the *Igh* locus, the chromatin landscape of the promoter, as well as of the RSS, contributes to Vκ gene recombination. Thus, multiple facets of local chromatin features explain much of the variation in Vκ gene usage. Together, these findings reveal shared and divergent roles for epigenetic features and transcription factors in AgR V(D)J recombination and provide avenues for further investigation of chromatin signatures that may underpin V(D)J-mediated chromosomal translocations.

## Introduction

V(D)J recombination enables the sequential rearrangement of variable (V), diversity (D) and joining (J) gene segments in B and T cell antigen receptor (AgR) loci. This mechanism, catalysed by the RAG recombinase complex which recognises the recombination signal sequence (RSS) of each gene segment, is the essential first step in the generation of diverse AgR repertoires, transforming a couple of hundred genes into millions of different antigen specificities (1). In B cells, the immunoglobulin heavy chain (*Igh*) locus recombines first, with D to J_H_ recombination on both alleles preceding V_H_ to DJ_H_ recombination on one allele in pro-B cells (2). The joining of these genes is imprecise, due to exonuclease activity and the addition of non-templated nucleotides, partly mediated by terminal deoxynucleotidyl transferase (TdT), thereby enhancing diversity (3). Functional IgH chains are expressed on the cell surface with the surrogate light chain as the pre-B cell receptor. This promotes proliferation, differentiation to the pre-B cell stage and recombination of the immunoglobulin kappa light chain locus (*Igκ*) (4, 5).

The mouse *Igκ* locus, located on chromosome 6, is 3.2 Mb in size and contains 162 Vκ genes, 5 Jκ genes and a single Cκ gene (6). In contrast to the *Igh* locus, in which all V_H_ genes are in the same orientation, over half of the Vκ genes are in the reverse orientation with respect to the Jκ and Cκ genes (6), and their recombination leads to inversion rather than to the deletion of the intervening DNA. Whilst joining is still imprecise, light chain V-J junctions are much less diverse than *Igh* junctions since TdT is not expressed in pre-B cells (7, 8) and exonuclease activity is reduced (9). Surface expression of IgH and Igκ together as the B cell receptor (BCR) allows selection that favours productive VκJκ rearrangements and eliminates autoreactive BCRs. If necessary, recombination between the remaining upstream Vκ and downstream Jκ genes, termed receptor editing, is permitted (10); rearrangement of the second allele may also occur. The first recombination event at each allele is biased towards usage of the *Jκ1* gene, through suppression of DNA breaks at downstream Jκ genes (11).

The RAG recombinase-recruiting RSS of each V gene varies in quality, which can be quantified as the RSS information content (RIC) score, with a higher score theoretically more conducive to recombination (12, 13). However, accumulating evidence shows that whilst the RIC score provides one layer of regulation, epigenetic features including H3K4 methylation also contribute to regulation of VDJ recombination (14–20). Moreover, several transcription factors (TFs), including PAX5, IRF4, IKAROS, PU.1, E2A and P300, promote the activation and recombination of the *Igκ* locus. However, their specific contribution to shaping the repertoire is unclear (21–29), and may include long-range or local V gene roles, or a combination thereof. Loss of CTCF or of its binding sites leads to increased transcription and usage of J-proximal V genes in the *Igh* and *Igκ* loci. This suggests a role in long-range looping of the locus, bringing the distal V genes into proximity with the (D)J region (30–34). Deletion of PAX5 or YY1 also reduces distal V_H_ gene recombination in the *Igh* locus (35, 36). However, these general biases towards proximal recombination cannot explain why genes that are close to each other and similar in sequence can recombine at substantially different levels. A recent RNA-based, high-throughput analysis of the expressed mouse Vκ gene repertoire revealed that it was highly variable across the locus (37). Similarly, a DNA-based assay revealed diverse but variable Vκ gene usage in mouse splenic B cells (38); both studies also revealed that the Vκ repertoire for each Jκ gene differed. Highly represented Vκ genes in the RNA repertoire interact more frequently with *Igκ* enhancers compared to genes represented at low levels, and E2A has been implicated in orchestrating these interactions (39, 40). YY1 may direct the recombination of specific Vκ genes since expression of a YY1 mutant lacking a Polycomb Group binding domain resulted in a skewed repertoire in mouse pre-B cells (41), although a concomitant decrease in receptor editing may contribute to this finding. Thus, the features of the *Igκ* locus that determine the capacity of each Vκ gene to recombine, and the nature of their contribution, are poorly understood.

We recently developed the DNA-based VDJ-seq assay for unbiased high-throughput quantitation of *Igh* V_H_ and D_H_ repertoires and applied it to mouse bone marrow pro-B cells (14). By integrating our VDJ-seq data with genome-wide datasets for numerous histone modifications and TFs, we identified two mutually exclusive chromatin states: an architectural state, characterised by binding of CTCF and RAD21, and an enhancer state, characterised by binding of IRF4, PAX5 and YY1 and by histone modifications associated with enhancers and transcriptional activation. These chromatin states form at the RSS of V_H_ genes, and both are highly predictive of active recombination (14). Moreover, they are enriched at non-canonical genome-wide binding sites for the recombinase enzymes that catalyse V(D)J recombination (42–44), suggesting these states may also be permissive for the aberrant recombination events that underpin B cell leukaemias. The extent to which the chromatin signatures that underpin V(D)J recombination are shared between AgR loci is unknown. Moreover, whether a consensus signature exists that is predictive of susceptibility to aberrant recombination remains poorly understood.

Whilst the expressed Vκ gene repertoire has been quantified in pre-B cells (37), this does not accurately reflect the comparative frequency of recombination of each gene at the DNA level. This is because RNA quantity is an indirect measure of recombination frequency and is affected by factors that include different Vκ gene promoter strengths, the ratios of productive (in-frame):non-productive rearrangements, and transcript stabilities. For example, many recombined Vκ pseudogenes will not be detected in an RNA-based assay. However, 11 pseudogenes were detected in the DNA repertoire of splenic B cells (38), and the frequency of these events is much higher in pre-B cells, before non-functional rearrangements have been removed (45, 46).

In this study, we adapt the VDJ-seq assay for the unbiased profiling and analysis of the VκJκ repertoire, and present a comprehensive inventory of Vκ gene usage in mouse bone marrow pre-B cells, the pre-selection population of B cells in which recombination is taking place. We additionally build a novel two-step machine learning model to study the relationship between the primary Vκ gene repertoire and locus-wide profiles of chromatin features and transcription. Our results both confirm previous findings concerning the potential mechanisms that underpin recombination of the *Igκ* locus, and substantially advance our understanding of these regulatory mechanisms. We found that local chromatin features are highly predictive of whether a given Vκ gene is recombined or not, and of its recombination frequency. In contrast to the *Igh* locus, we observed that not only the RSS but also the Vκ gene promoter and its surrounding chromatin contribute to recombination frequency. We identified PU.1 binding at the RSS as a crucial feature in determining whether or not a Vκ gene will actively recombine, whilst IKAROS binding and H3K4 methylation are important in promoting a higher frequency of recombination. Moreover, whilst some local chromatin features that drive recombination are shared with the *Igh*, the regulatory mechanisms contributing to recombination of these two AgR loci are substantially different.

## Materials and Methods

### Primary Cells

C57BL/6 (WT) and *Rag1^–/–^/VH81X* mice were maintained in accordance with local and Home Office rules and ARRIVE guidelines under Project Licence 80/2529.

For each biological replicate, bone marrow from nine 6- to 8-week-old *Rag1^−/−^/VH81X* mice (47, 48) or from fifteen 12-week-old male wild-type (WT) C57BL/6 mice was incubated with biotinylated antibodies against CD11B (MAC-1; ebioscience), Ly6G (Gr-1; ebioscience), Ly6C (Abd Serotec), TER119 (ebioscience), and CD3E (ebioscience) followed by incubation with streptavidin MACs beads (Miltenyi), to deplete macrophages, granulocytes, erythroid lineage, and T cells. Pre-B cells (surface IgM*^−^*CD25^+^B220^+^CD19^+^) were then flow sorted on a BD FACSAria in the Babraham Institute Flow Cytometry facility. Antibodies used were CD45R BV421 (B220, RA3-6B2, Biolegend), CD19 PerCP-Cy5.5 (1D3, BD Pharmingen), CD25 APC (PC61.5, eBioscience), and IgM PE (eB121-15F9, eBioscience). Sort purities were all greater than 92%.

### VDJ-Seq

#### VκJκ-Seq Assay

The VDJ-seq assay (14) was adapted for analysis of the *Igκ* repertoire (Supplementary Text S1 and Figure S1 in Supplementary Material). DNA was isolated from flow sorted pre-B cells using a DNeasy kit (Qiagen) and 10 µg was sonicated to 400 bp using a Covaris E220 sonicator. Except where AMPure XP beads were used, all following reactions were cleaned up by column purification (Qiagen QIAquick PCR purification kit). Fragmented DNA was end-repaired and A-tailed using standard protocols. Samples were divided in half and short asymmetric adaptors, including a molecular identifier and one of the two different anchor sequences, were ligated to both ends of all fragments (T4 DNA ligase, NEB; 16°C overnight); the two ligations were then pooled. Primer extension (8 µl × 50 µl; 2 U NEB Vent Exo-polymerase per reaction) using biotinylated primers that anneal downstream of all functional Jκ genes (Jκ1, 2, 4, and 5) allowed for the enrichment of fragments that contain a Jκ gene using streptavidin beads (My-one C1; Invitrogen), following the manufacturer’s protocol with incubation overnight, rotating at room temperature (20 µl beads per sample). After washing the beads, four cycles of PCR amplification off the beads were performed, using an Illumina PE1 primer corresponding to the long strand of the asymmetric adaptors, in combination with Jκ-specific PE2 primers (4 µl × 25 µl; Pwo master mix, Roche). A second primer extension reaction (4 µl × 50 µl) using biotinylated primers that anneal within intergenic regions upstream of each functional Jκ gene (i.e., present only when unrecombined) was then performed. Unrecombined sequences were removed using streptavidin beads with a 4 h incubation at room temperature. The remaining DNA fragments, containing the Vκ-Jκ recombined sequences, were further enriched, with 11 additional PCR cycles using the same PE1/Jκ-PE2 primers as above. PCR products were cleaned up and small products removed, using AMPure XP beads (1×; Beckman Coulter). A final PCR amplification of five cycles was performed to add the flowcell-binding portions of the PE1 and PE2 adaptors, including Illumina Truseq bar codes within PE2. Final libraries were purified and size-selected by double-sided AMPure XP bead purification (0.5× followed by 1×), before quality control using a high sensitivity DNA assay on the Agilent Bioanalyzer, and KAPA qPCR (Illumina library quantification kit, KAPA Biosystems). Libraries were sequenced on the Illumina HiSeq, with 2 × 100bp paired end sequencing. Sequences of all oligonucleotides used and the cycling conditions are provided in Table S4 in Supplementary Material.

#### VκJκ-Seq Pipeline

We adapted our Babraham LinkON pipeline (14)^1^ for processing of VκJκ-seq data. Briefly, sequences were demultiplexed based on Truseq barcodes and trimmed for adaptors and low quality (Phred < 20) using TrimGalore version 0.3.8 (Babraham Bioinformatics^2^). Due to the similarity of the J primers, the sequencing quality can drop at positions 3–4 for the J reads (Read 2). The first four bases were, therefore, trimmed off all J read sequences (using the option—clip r2 4 in Trim Galore). Chimaeric J reads produced through mis-priming of a Jκ gene with the incorrect Jκ-PE2 PCR primer were identified by examining the sequence immediately downstream of the primer binding sites, and a find-and-replace step was used to replace the incorrect Jκ primer sequences within commonly occurring chimaeras with the correct primer sequence. Thereafter, the Jκ primer (“bait”) sequences were used to assign each read to the corresponding Jκ gene. Any sequence without a bait was discarded. Sequences were further filtered to exclude any that had less than 20 bases downstream of the bait in Read 2, or that did not include one of the two anchor sequences following the molecular identifier in Read 1. The V end reads (Read 1), excluding the first 15 bases, which comprise the molecular identifier and anchor sequence, were aligned to the NCBIM37/mm^9^ mouse genome assembly using Bowtie version 1.1.0 (49), discarding multi-mapping hits (options: “-m 1—strata—best”). The data were de-duplicated based on: the sequence of the molecular identifier (6N); the sequence downstream of the bait in Read 2, which includes the Vκ–Jκ junctions; and the start position of the V read alignment. Any paired reads identical for all criteria were considered to be PCR duplicates, and only one was retained. Finally, aligned Read 1 sequences were produced as output as Bowtie mapped unique_V-BAM files. The entire pipeline is documented in more detail here: https://github.com/FelixKrueger/BabrahamLinkON/blob/master/run_VkSk-Seq_pipeline.md. Read counts for each stage of the pipeline are shown in Figure S2A in Supplementary Material.

### Quantification of Vκ-Jκ Recombination

BAM files of aligned V reads were loaded using default parameters into Seqmonk version 1.37.1 (Babraham Bioinformatics^3^), a tool for the visualisation and analysis of next generation sequencing data. Based on examination of the reads, two Vκ gene annotations were corrected (Supplementary Text S2). V reads on the same strand as the gene were then counted within windows extending 750 bp upstream (with respect to the gene orientation) from the 3′ end of each Vκ gene, for each Jκ gene separately. Read counts were normalised to the replicate with the median number of reads (replicate 1), either across all Js, or just for Jκ1-associated reads. For Jκ1-associated V sequences, the median of the normalised replicates for each Vκ gene was used as the recombination frequency in downstream analyses. The mappability (defined as the percentage of all possible 85 bp sequences over a given window that can be mapped uniquely) over 350 bp windows upstream of each Vκ gene 3′ end (within which the vast majority of VκJκ-seq reads are localised) was calculated, and where stated, genes with low mappability (below 70%) were excluded. Only 11 genes fell into this category, 10 of which actively recombine, and only 4 of these had mappability below 60%. Quantitated data and other information relating to each Vκ gene is provided in Table S1 in Supplementary Material.

### IMGT HighV-QUEST Analysis

To facilitate analysis by IMGT HighV-QUEST, a tool for the high-throughput analysis of V(D)J-recombined sequences (50), V and J sequences were first merged, and any gaps filled in, as described previously (14). *Igκ* rearrangements were then analysed by IMGT HighV-QUEST, with default parameters. Complementarity determining region 3 (CDR3) lengths and productive versus non-productive rearrangement data were obtained from the “Summary” file.

### Definition of Active and Inactive Genes

We defined active genes as those that were significantly enriched (padj < 0.01) for VκJκ-seq reads compared to the Vκ region as a whole, using a binomial test. Thus, the probability was defined as the length of the window in which reads were quantified, as a fraction of the total length of the Vκ region, *n* as the rounded median of normalised read counts for each gene, and *N* as the rounded median of normalised read counts across the entire Vκ region.

### ChIP Co-Localisation and Enrichment Analysis

ChIP peaks (including DHS) were called using MACS peak calling algorithm (51). MACS 1.4, which performs better with broad peaks was used for H3K27me3 with pvalue cutoff = 1.00e−02. For all the remaining features, MACS2 was used with pvalue cutoff = 1.00e−05. The number of peaks over the *Igκ* locus for each dataset is shown in Table S3 in Supplementary Material.

For each Vκ gene, we calculated the distance from the centre of the gene to the closest upstream and closest downstream (defined with respect to the gene orientation) peak summits. If a peak summit was located upstream of the gene centre, and within 1 kb of the gene start site, it was labelled as promoter-associated; conversely any peaks with summits located downstream of the gene centre and within 1 kb of the gene 3′ end were labelled as RSS-associated.

Relative enrichment for ChIP-seq datasets was calculated as the number of reads over a given window, relative to the average number of reads within windows of identical size across the entire *Igκ* locus.

### Phylogenetic Analysis

A phylogenetic tree of C57BL/6 mouse Vκ gene germline sequences, based on NCBI Reference Sequence: NG_005612.1^4^ but with the corrected annotations detailed in Supplementary Text S2, was constructed using the Phylogeny.fr tool, without including alignment curation (52, 53). Multiple sequence alignment was performed with MUSCLE (54), and the maximum likelihood used for tree construction (55, 56). The tree was visualised using the R package ggtree (57), and nodes comprising the majority of each Vκ gene family were collapsed.

### Computational Approach

Our computational approach comprises an unsupervised and a supervised step. In the unsupervised step, we set out to interrogate the chromatin landscape of the *Igκ* locus through integration of the histone modification, DNase hypersensitivity (DHS), germline transcription, and TF-binding profiles included in this study. The supervised step is constructed in two layers: first, we train a Random Forest Classifier (RF-C) C(X) to predict whether a given gene is active or not; second, we construct a Random Forest Regression (RF-R) model R(X) to predict the frequency of recombination of a given active gene. In what follows, we describe both steps in more details.

#### Chromatin Segmentation Analysis

For the supervised step, we used EpiCSeg (58), which combines the input features for the segmentation and characterisation of a context-specific chromatin landscape. EpiCSeg was originally developed to learn the epigenomic landscape from histone marks. However, we chose this algorithm instead of its commonly used counterparts, such as chromHMM (59), which works well for combinations of TFs and histone marks. This was because while the underlying mathematical modelling is very similar, it bypasses the binary mode of chromHMM and allows the user to proceed with continuous values, preventing loss of information and overfitting, which is particularly useful for analysis of a single large locus.

For this, we divided the locus into 200 bp non-overlapping bins and calculated the enrichment of each feature over all bins using bedtools (60) multibam coverage function. As an input for EpiCSeg, we constructed a raw read-counts matrix X in which x_{ij} corresponds to the enrichment of feature i in bin number j. We ran EpiCSeg with varying numbers of states ranging from 3 to 15.

The A or E state were assigned to the promoter or RSS, if they overlapped a window extending from the centre of the gene to 500 bp up- or downstream, respectively, with the exception that if the A/E state segment did not overlap with the gene start, and its centre was downstream of the gene centre, it would be assigned to the RSS but not the promoter, or vice versa.

### Random Forest Classification and Regression Models

For the unsupervised step, we first trained a RF-C to predict whether a Vκ gene is “active” or not. We then constructed a RF-R model to predict the recombination level of an active gene. We chose RF since it is generally accepted to be superior in tackling high dimensionality (relatively high number of features with low number of samples for the training) and co-linearity between the features (61).

Read counts for DHS-seq, ChIP-seq, and RNA-seq data were generated using Seqmonk, within four distinct windows for each Vκ gene: “promoter,” extending from 500 bp upstream of the start of the gene to its centre; “RSS,” extending from the centre of the gene to 500 bp downstream of its 3′ end; and “upstream” and “downstream” windows, extending from 500 bp to 3 kb up- or downstream of the gene start or end, respectively. In addition to these four windows for each of the genome-wide datasets, giving a total of 76 chromatin features, we also included three genetic features. These were: the RSS RIC score, the orientation (or strand) of the Vκ gene, and the distance from the Vκ gene to Jκ1. All of these features, except for the gene orientation, were projected between 0 and 1. These 79 features were considered as the explanatory variable for both the RF-C and RF-R.

The response variable for RF-C was the binary recombination classes (active and inactive), which were defined as described above. For RF-R, the log2-transformed median of the normalised recombination frequencies of active genes was used as the response variable.

Both the RF-C and RF-R approaches were performed with 10-fold cross-validation: 10% of genes were assigned to the test set each time, with every gene included in a test set exactly once. The number of trees generated for each fold was 1,000. For the initial RF-C including all features, the number of features tried at each step was set to 20; for all other models default parameters were used. The average importance of each feature, and SE across the 10-folds, was recorded. For the classification model, the performance was assessed by calculating the percentage of correct predictions (accuracy) across all ten test sets: this was calculated overall, as well as for the active Vκ genes (giving a measure of the sensitivity with which we could identify an active gene) and inactive genes (which gives a measure of specificity). We also calculated the F1 score as a combined measure of sensitivity and specificity. To assess the performance of the regression model, we used the root mean squared error (RMSE) for the predicted recombination frequencies compared to the observed values across all ten test sets. The RMSE gives a measure of the SD in errors, thus 68% of our predictions are expected to have an error within this range. Since our recombination frequencies are log2-transformed, an RMSE of *x* corresponds to a 2*^x^*-fold difference between the predicted recombination frequency and the observed recombination frequency.

For model selection, we focussed on the 16 most important features from the initial classification or regression model. We trained RF classification or regression models, with 10-fold cross-validation, for all possible combinations of the respective 16 features. These models were then compared using the performance metrics described above. Our analysis was performed using the R package randomForest (62).

### Data Availability

Publicly available genome-wide datasets analysed during this study are available in the GEO repository; details including accession numbers are listed in Table 1. All were downloaded from GEO as raw short-read files (SRA) and realigned to NCBIM37/mm9 using Bowtie (49) or Bowtie 2 (63). The VκJκ-seq datasets generated in this study are available in the GEO repository with accession number GSE101606.^5^ Some of the quantitated data from this study is also provided in Table S1 in Supplementary Material.

**Table 1:** Publicly available next generation sequencing datasets utilised in our study.

Feature	Reference	Series	Accession(s)	Bowtie settings and notes
Nuclear RNA	Bolland et al. (14)	GSE80155	GSM2113570	bowtie -n 0 -m 1—best—strata— maxins 1,000
DHS (DNaseI hypersensitivity)	Revilla-i-Domingo et al. (64)	GSE38046	GSM932968	Expt. 8,439 (1). bowtie -m 1—best—strata
H3K4me1	Revilla-i-Domingo et al. (64)	GSE38046	GSM932934	Expt. 8,666 (1). bowtie -m 1—best—strata
H3K4me2	Lin et al. (39)	GSE40173	GSM987804	bowtie -m 1—best—strata
H3K4me3	Bolland et al. (14)	GSE80155	GSM2113571, GSM2113573	bowtie -n 0 -m 1—best—strata—maxins 700
H3K9ac	Revilla-i-Domingo et al. (64)	GSE38046	GSM932943, GSM932944, GSM932945, GSM932946	Expts. 8,108, 8,113. bowtie -m 1—best—strata
H3K27me3	Revilla-i-Domingo et al. (64)	GSE38046	GSM932947, GSM932948, GSM932949, GSM932950, GSM932951	Expts. 8,111, 8,116. bowtie -m 1—best—strata
CTCF	Ebert et al. (65)	GSE27214	GSM672401	bowtie -m 1—best—strata
RAD21	Ebert et al. (65)	GSE27214	GSM672403	Bowtie -m 1—best—strata
P300	Lin et al. (39)	GSE40173	GSM987808	bowtie -m 1—best—strata
PAX5	Revilla-i-Domingo et al. (64)	GSE38046	GSM932924	Expt. 8,417. bowtie -m 1—best—strata
YY1	Medvedovic et al. (66)	GSE43008	GSM1145864	bowtie -m 1—best—strata
PU.1	Mullen et al. (67)	GSE21614	GSM539538	bowtie -m 1—best—strata
MED1	Whyte et al. (68)	GSE44288	GSM1038263	bowtie -m 1—best— strata
EBF1	Vilagos et al. (69)	GSE35857	GSM876622, GSM876623	bowtie -m 1—best—strata
IRF4	Schwickert et al. (70)	GSE53595	GSM1296534	Bowtie2
E2A	Lin et al. (71)	GSE21978	GSM546523	Bowtie -m 1
BRG1	Bossen et al. (72)	GSE66978	GSM1635413, GSM1635414	Bowtie -m 1—best—strata
IKAROS	Bossen et al. (72)	GSE66978	GSM1635411, GSM1635414	Bowtie2

## Results

### VκJκ-Seq—A High-Throughput Assay for Quantification of Recombined Vκ Gene Repertoires

To quantify the usage of Vκ genes in an unbiased way from DNA, we adapted our previously reported mouse *Igh* VDJ-seq assay (14) for the mouse *Igκ* locus (Figure S1 and Supplementary Text S1 in Supplementary Material). We generated VκJκ-seq data for three biological replicates in wild-type (WT) bone marrow pre-B cells (B220^+^/CD19^+^/CD25^+^/IgM^−^), and one replicate in pre-B cells from a *Rag1*^−/−^ mouse with a rearranged *Igh* transgene (*VH81X*) (47). These *Rag1*^−/−^*/VH81X* cells lack the RAG1 recombinase, precluding V(D)J recombination, but progression to the pre-B cell stage is permitted through expression of the *VH81X* transgene. Thus, they serve as a negative control in which reads mapping to Vκ genes, indicating a VκJκ recombination event, should not be detected, giving a measure of the spurious incorporation of these reads into our libraries. Indeed, while 91.8% of unique, Jκ bait-associated reads for this library mapped upstream of unrecombined Jκ genes, only 9 reads (0.0002%) mapped to Vκ genes. Conversely, in WT pre-B cells over 30% of reads mapped to Vκ genes for all replicates. This equated to a total of 400–530,000 unique VκJκ recombined fragments for each replicate (Figure S2A in Supplementary Material), an order of magnitude greater than previous high-throughput assays of the Vκ gene repertoire (37, 38). In the *Rag1*^−/−^*/VH81X* library, we noted a slight bias towards *Jκ2* (Figure S2B in Supplementary Material), suggesting minor preferential priming of the *Jκ2* gene. However, within the VκJκ recombined fragments, over 35% of reads were associated with *Jκ1*, which usually recombines first (11), indicating that we are capturing a large proportion of the primary Vκ gene repertoire. Analysis of the sequences using IMGT/HighV-QUEST revealed that the ratio of productive:non-productive rearrangements was approximately 37:63 (Figure S2C in Supplementary Material), close to the expected two-thirds of non-productive rearrangements. Consistent with previous reports (8, 9, 38), the vast majority of functional rearrangements had a CDR3 of 9 amino acids (Figure S2D in Supplementary Material).

**Figure 1:**
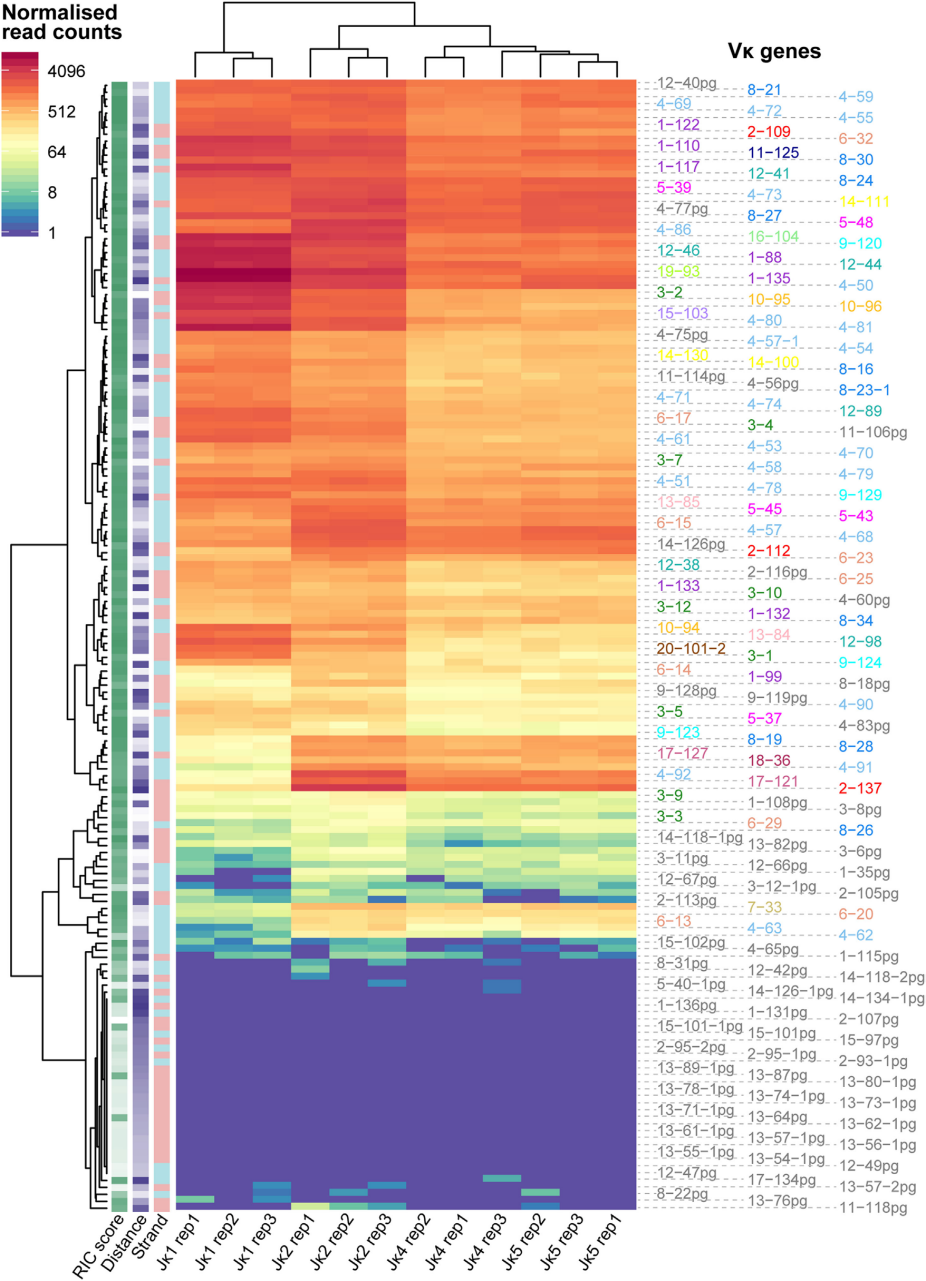
Recombination frequencies for all Jκ genes and replicates. Heatmap showing log2-transformed recombination frequencies for each Vκ-Jκ combination across all replicates. Read counts for each replicate were first normalised to the replicate with the median number of reads aligning to Vκ genes. Each row represents a Vκ gene and each column represents an individual replicate for a Jκ gene. Vκ genes and Jκ gene replicates are clustered based on the similarity of their repertoires. The strand (+, pink; −, blue), recombination signal sequence (RSS) Information Content (RIC) score (low = light; high = dark) and distance from Jκ1 (low = light; high = dark) for each Vκ gene are displayed on the left, and the colour of the Vκ gene label represents its family.

Using normalised frequencies for each Vκ-Jκ gene combination, we clustered the dataset based on both Vκ and Jκ genes, taking each replicate separately. While Vκ genes with poor RIC scores clearly recombine infrequently, we did not observe any relationship between the repertoire of Vκ genes and their distance from, or orientation with respect to, the Jκ genes. For each Jκ gene, the three replicates were highly correlated (Pearson correlation coefficients >0.992) and clustered more closely to each other than to the repertoires of other Jκ genes, indicating that they are associated with distinct Vκ gene profiles (Figure 1). Notably, the pattern of recombination to *Jκ1* segregated from the other Jκ genes. This is consistent with the preferential usage of *Jκ1* in the generation of the primary repertoire, while the other Jκ genes are subsequently used for receptor editing (11). Since we aimed to assess the features driving recombination of the germline *Igκ* locus in the formation of the primary repertoire, we chose to focus on the *Jκ1* repertoire for further analyses (Table S1 in Supplementary Material).

**Figure 2:**
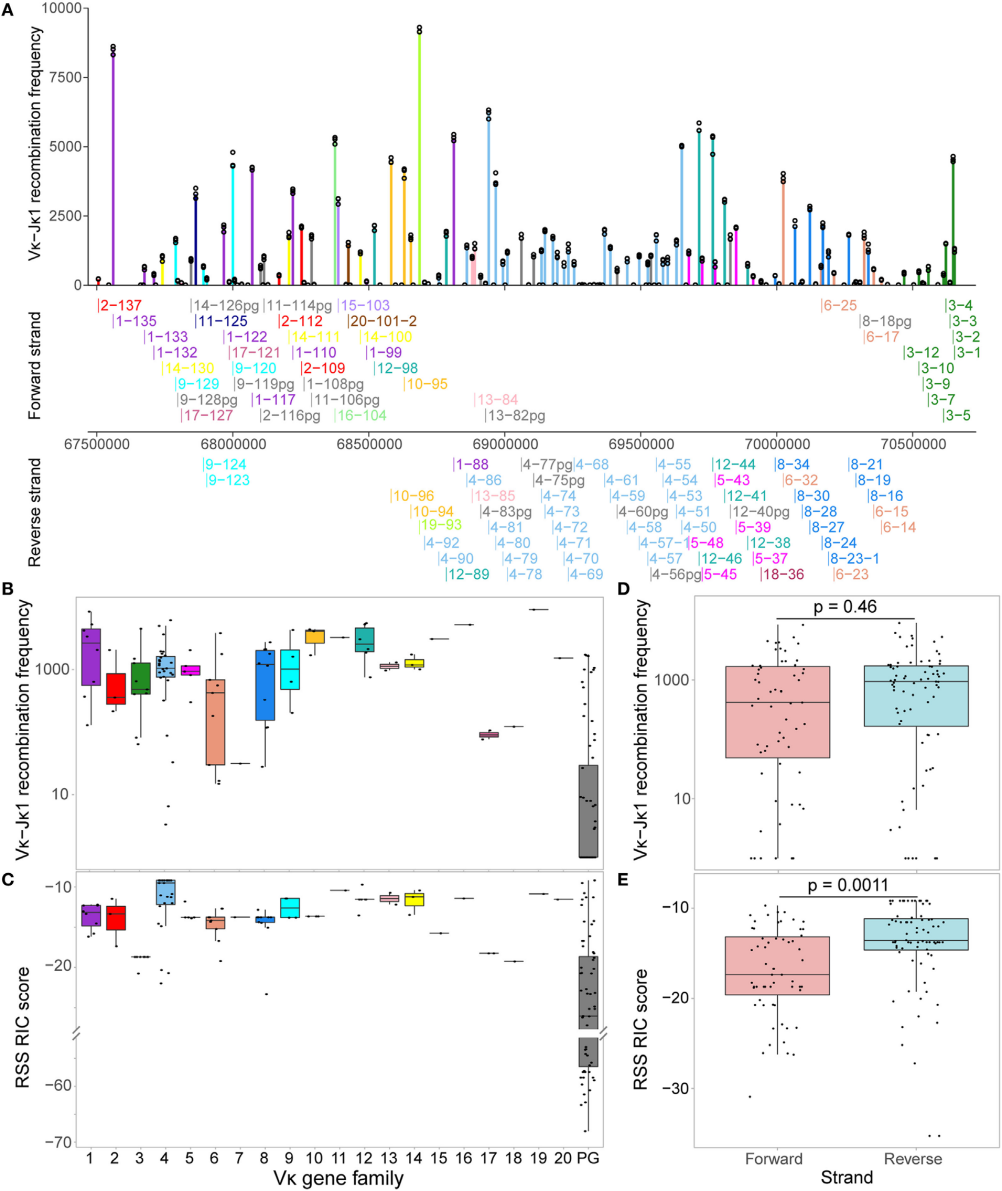
Vκ recombination to Jκ1 varies widely across the locus. **(A)** Reads associated with Jκ1 for each Vκ gene were counted for all replicates, and normalised to the replicate with the median total number of Jκ1-associated Vκ reads. Bars represent the median normalised read count, while each replicate is displayed as a circle. Genes are arranged geographically, from the 5′ end of the locus (left) to the 3′ end (J-proximal end; right). Below, names and localisation on chromosome 6 of all actively recombining Vκ genes (Figure 3A) are shown, with genes on the forward strand (that recombine by deletion) displayed above the scale bar, and genes on the reverse strand (that recombine by inversion) displayed below. Vκ gene families are represented by colour, with pseudogenes (pg) displayed in grey. **(B,C)** Normalised median recombination frequencies to Jκ1 **(B)** and recombination signal sequence (RSS) Information Content (RIC) scores **(C)** are shown for all genes in each Vκ family. The 64 pseudogenes (PG) originate from 13 out of the 20 Vκ gene families. **(D,E)** Normalised median recombination frequencies **(D)** and RSS RIC scores **(E)** for genes on the forward and reverse strands. Only genes with a RIC score > −38.81, which are considered theoretically capable of recombination, are included; *p*-values from two-sided *t*-tests are shown.

The Vκ-Jκ1 repertoire varied widely across the locus, with no clear geographical pattern (Figure 2A). The RIC scores of Vκ genes from the same family were quite homogeneous, while their recombination frequencies could vary by more than 10-fold (Figures 2B,C). Comparing the families, we noted some patterns: for example, Vκ1 genes recombine quite frequently compared to several other families, even when their median RIC score was similar (e.g., Vκ2) or higher (e.g., Vκ4). For all genes with a RIC score > −38.81, which are theoretically considered capable of recombination (12), usage of Vκ genes on the forward and reverse strands was not significantly different (Figure 2D), despite the significantly lower RIC scores of forward compared to reverse strand genes (Figure 2E). This contrasts with observations from an RNA-based assay (37) in which recombination to *Jκ1* was biased towards inversional rearrangements. Moreover, while 6 out of the 10 Vκ genes that were most frequently represented in their expressed *Jκ1* repertoire (37) are included in the top 20 of our Vκ-Jκ1 repertoire, 4 are not, and the DNA repertoire is not dominated by a small number of genes. Our assay also reveals numerous Vκ genes that are more highly represented at the DNA level, including 14 pseudogenes that were not detected in the RNA repertoire. Conversely, all Vκ genes present in the expressed repertoire were detected in our assay, albeit in some cases with very low read counts. This highlights the significant contribution of transcription and posttranscriptional processes to the expressed repertoire, which would confound the aim of this study to interrogate the pre-recombination chromatin state.

**Figure 3:**
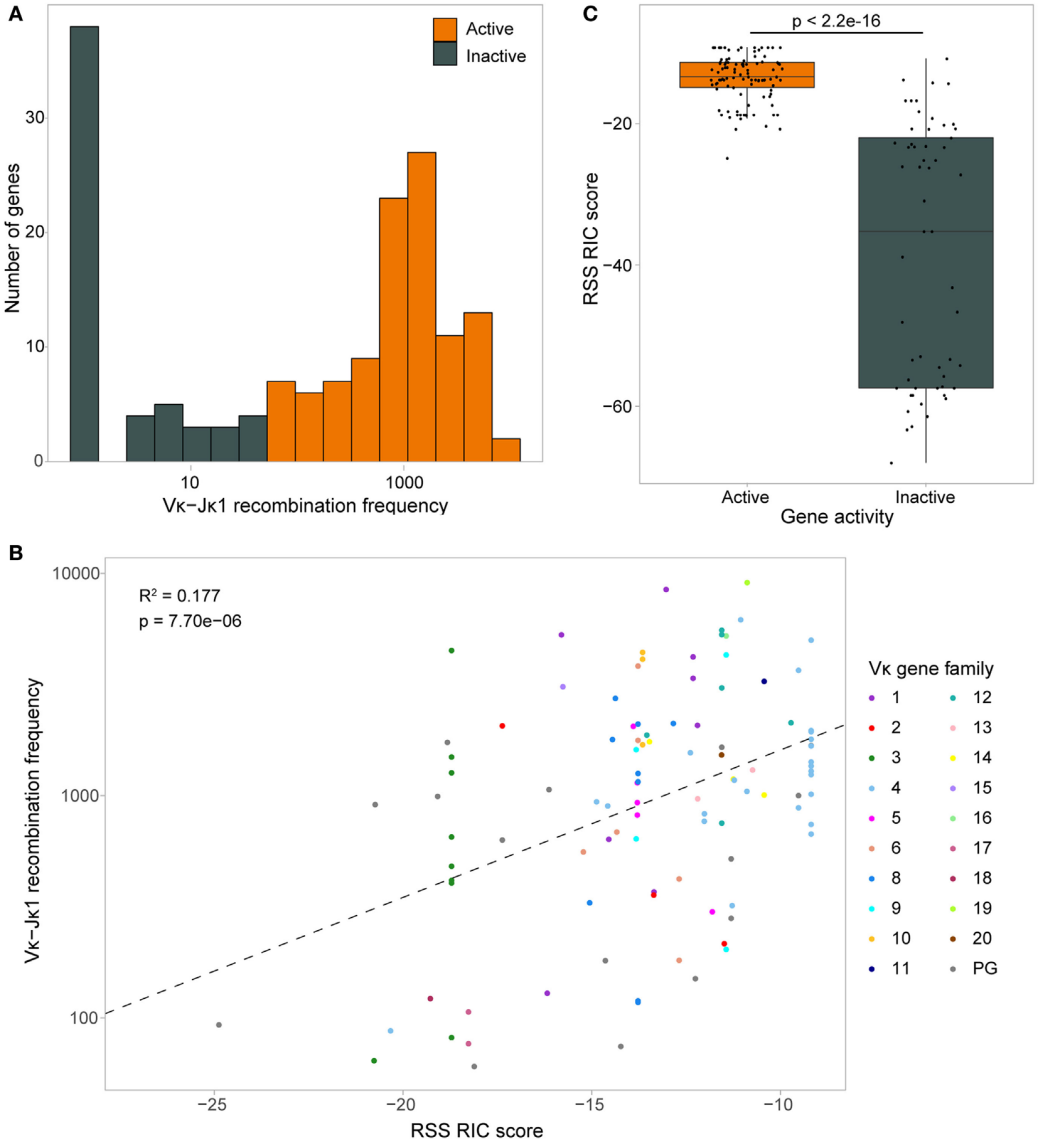
Recombination signal sequence (RSS) Information Content (RIC) score can only partially explain variation in Vκ gene activity. **(A)** Distribution of Vκ-Jκ1 recombination frequencies for all 162 Vκ genes. A one-sided binomial test was used to gauge the significance of their recombination frequency, allowing each gene to be labelled as active (fdr adjusted *p*-value <0.01) or inactive. **(B)** Dependence of active genes’ recombination frequency on RSS RIC score. Linear regression model (dashed line) reveals that only 17.7% of the variation in recombination frequency of active genes can be explained by the RIC score. **(C)** RSS RIC scores of active and inactive Vκ genes; *p*-value from a two-sided Wilcoxon rank sum test.

To facilitate further investigation of the Vκ-Jκ1 repertoire, we performed a binomial test to distinguish Vκ genes that are significantly recombining (padj < 0.01, Figure 3A; Table S1 in Supplementary Material). Out of 162 genes, 105 (64.8%) passed the binomial test and were labelled “active” to denote “actively recombining”; these genes were detected with a minimum of 59 reads, and included 15 pseudogenes, and are hereafter referred to as active genes. The remaining 57 genes had insufficient evidence of activity, with the median read count for each below 39, and were labelled “inactive.” These inactive genes included eight Vκ genes that are considered to be functional, suggesting that they contribute little to the primary repertoire. The usage of active genes was weakly correlated with the RIC score (*R* = 0.42; Figure 3B); however, genes with a similar RIC score could recombine at markedly different frequencies. Moreover, some inactive genes have RIC scores that are comparable to those of active genes (Figure 3C). Importantly, a linear regression model revealed that only 17.7% of the variability in Vκ gene usage could be explained by RIC score alone (Figure 3B), highlighting the need to explore whether other mechanisms, such as chromatin features, contribute to shaping the repertoire.

### Chromatin Landscape of the *Igκ* Locus

#### Colocalisation of Chromatin Features with Vκ Genes

In order to assess the contribution of chromatin features to Vκ gene recombination, we used published genome-wide datasets from mouse pro-B cell models that are developmentally stalled prior to recombination of the *Igh* locus (48, 73). There are numerous pro-B cell datasets available, and the regulatory state of the *Igκ* locus has already begun to be established by this stage (39, 40, 74). Our analysis aims to determine the importance of these early regulatory events in priming the locus for recombination, thus shaping the primary repertoire. Moreover, *Igκ* locus gene-specific studies (75, 76), as well as the small number of available pre-B cell datasets (32, 77), revealed similar enrichment of CTCF, YY1, and histone H3 acetylation in pro-B and pre-B cells. The chromatin features we chose to assess included DHS, germline transcription, and ChIP for several histone modifications and TFs (Table 1).

**Figure 4:**
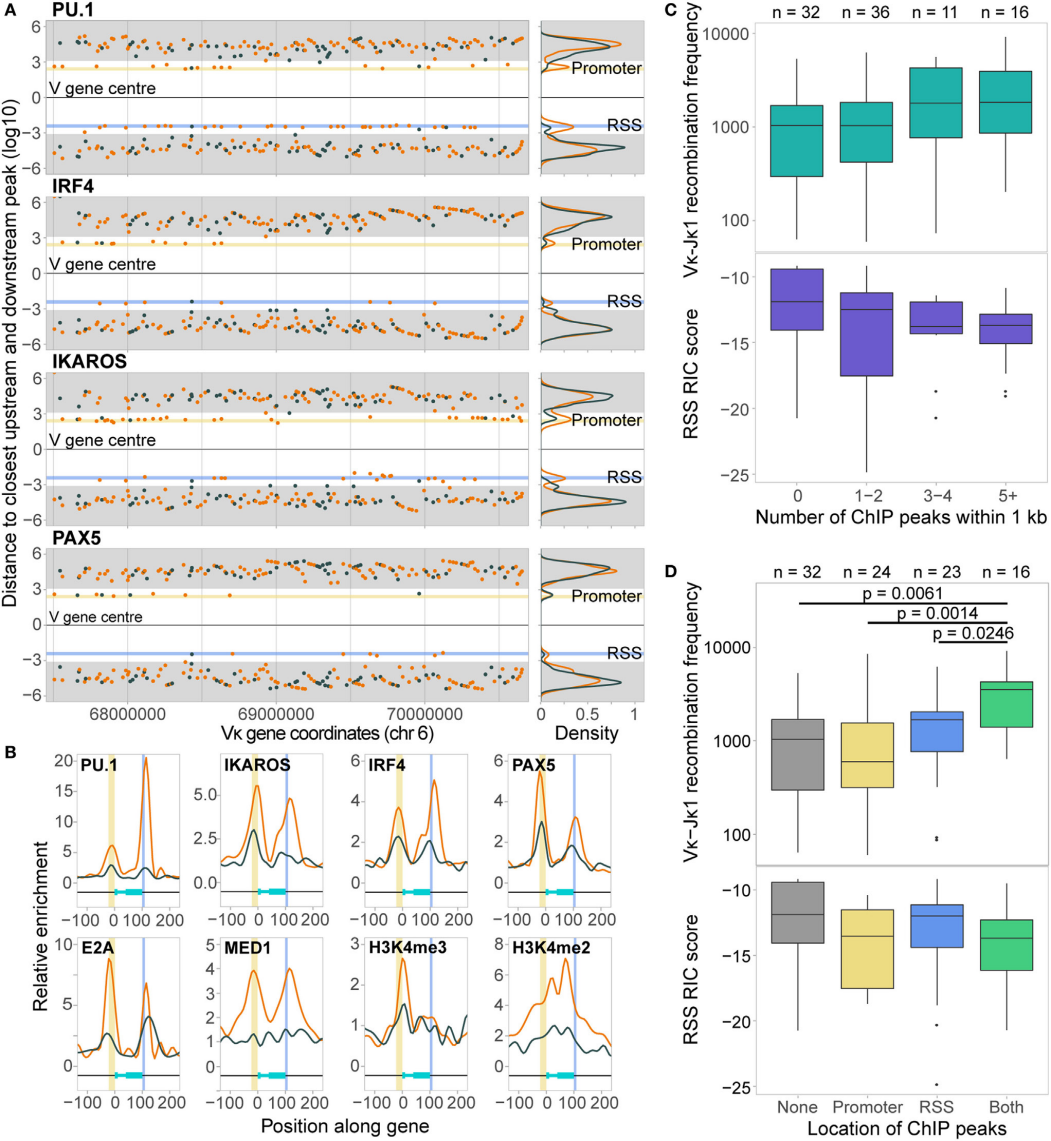
Chromatin features associate with both the promoters and recombination signal sequences (RSSs) of active Vκ genes. **(A)** Scatter plots and density plots showing the log10-transformed distances of the closest ChIP-seq peaks both up- (+) and downstream (−) from the centre of active (orange) and inactive (dark grey) Vκ genes. Yellow (promoter) and blue (RSS) shading indicates the range of distances within which 80% of the start and end sites of Vκ genes, respectively, are located. Grey shading indicates a distance of >1 kb from the Vκ gene, based on the median Vκ gene length (525 bp). **(B)** Average enrichment, relative to background, of chromatin features across all active (orange) and inactive (dark grey) Vκ genes. Genes have been scaled such that 0 and 100 represent the start and end of the gene, respectively. Yellow and blue shading indicates the location of the promoters and RSSs, respectively. **(C,D)** Median recombination frequencies and RSS Information Content (RIC) scores of active genes, excluding 10 genes that had <70% mappability. Genes are categorised based on the number of ChIP-seq peaks within 1 kb of the gene **(C)**, or the localisation of those peaks upstream (promoter) or downstream (RSS) of the gene centre **(D)**. *p*-values for Vκ-Jκ1 recombination frequency are fdr adjusted, based on a two-sided Wilcoxon rank sum test. *n* values indicate the number of genes in each category.

We first measured the distance from the centre of each Vκ gene to the summit of the closest peak for each DHS- and ChIP-seq dataset that had at least 35 peaks over the locus, both upstream (towards the promoter) and downstream (towards the RSS). Several TFs showed a bimodal distribution both up- and downstream of the Vκ genes. This was generally more pronounced for active Vκ genes, with peaks close to both promoters and RSSs (Figure 4A; Figure S3A in Supplementary Material). For some TFs, including PAX5 and IRF4, promoter-associated peaks were primarily located towards the 5′ end of the Vκ region, while PU.1 and IKAROS were also located close to promoters towards the 3′ end. Very few ChIP-seq peaks were found close to central Vκ gene promoters (Figure 4A). In contrast, RSS-associated peaks were located at Vκ genes throughout the locus. With the exception of PU.1, peaks were more frequently associated with Vκ gene promoters than with RSSs. We also noted that whilst RAD21 peaks directly mapped to only one promoter and one RSS, several peaks were located approximately 2 kb upstream of Vκ gene promoters (Figure S3A in Supplementary Material).

The localisation of TF peaks close to both the promoters and RSSs prompted us to examine the distribution of chromatin features over the Vκ genes in more detail, considering the overall enrichment of each feature without the threshold applied in peak calling. All TFs were found to be enriched over Vκ gene promoters, and most were enriched over RSSs, while the distribution of histone modifications was more variable (Figure 4B; Figure S3B in Supplementary Material). Importantly, with the exception of H3K27me3 and CTCF, the enrichment of all of these chromatin features was greater over active compared to inactive genes. Active genes with associated ChIP-seq peaks tended to recombine more frequently, despite having poorer quality RIC scores, than those without (Figure 4C), although this was not significant. Genes with peaks close to both the promoter and the RSS recombined with significantly greater frequency than genes with no associated peaks (*p* = 0.0061; Wilcoxon rank sum test) or with peaks that were only associated with the promoter (*p* = 0.0014) or RSS (*p* = 0.0246; Figure 4D). This suggests that both the promoter and the RSS are important in facilitating efficient recombination. This is in contrast to the *Igh* locus, in which TF enrichment is almost exclusively confined to the V_H_ gene RSSs (14), indicating that the mechanisms that regulate V(D)J recombination differ between V_H_ and Vκ.

#### Chromatin Segmentation of the *Igκ* Locus

In order to shed further light on how chromatin features contribute to Vκ gene recombination, we investigated the regulatory landscape of the *Igκ* locus with EpiCSeg (58). This algorithm employs a multivariate Hidden Markov Model to integrate genome-wide datasets and segment a given genomic locus into characteristic chromatin states. We used read counts over 200 bp bins covering the locus for each DHS- and ChIP-seq dataset as the input and ran the algorithm specifying an output of between 3 and 15 states.

**Figure 5:**
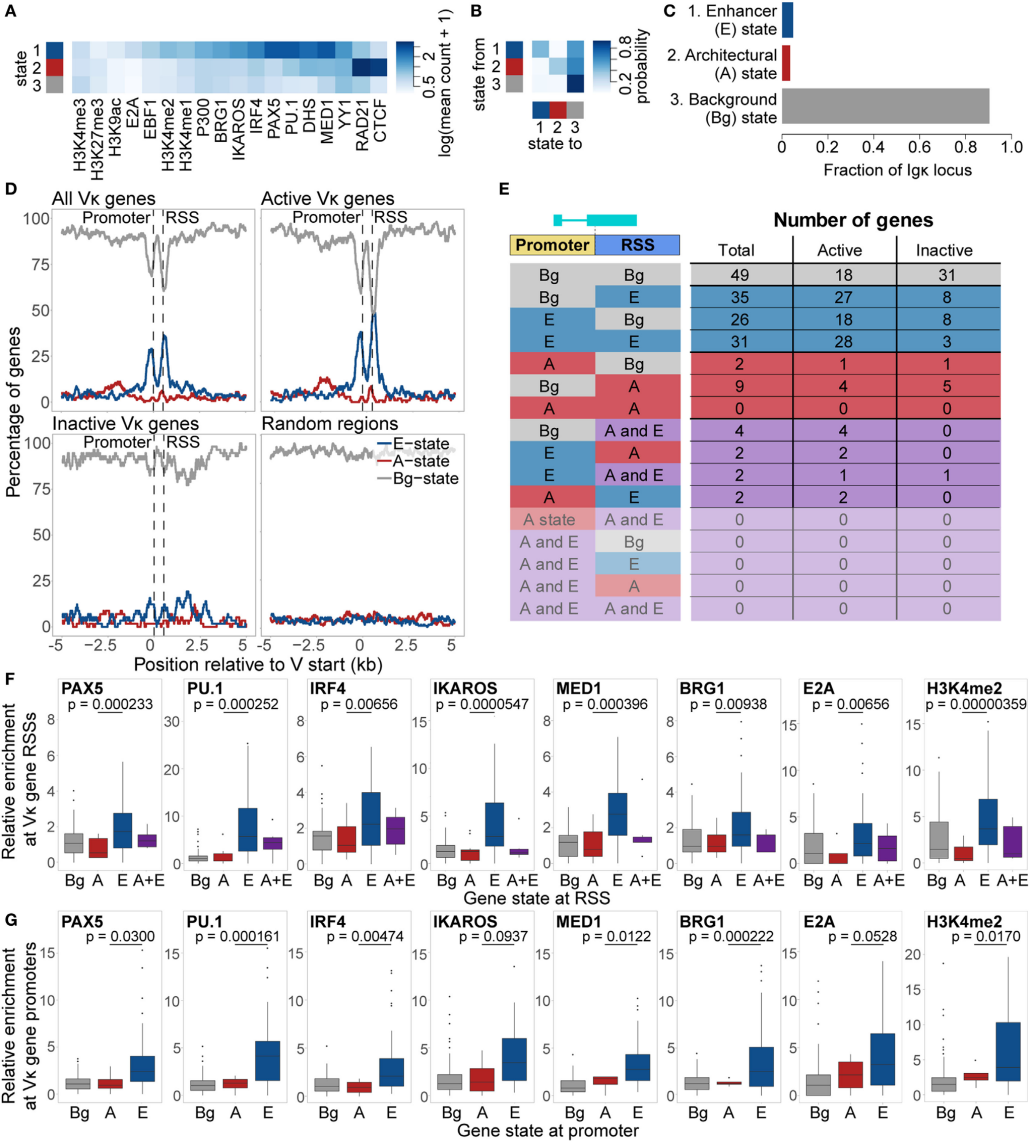
Chromatin state analysis of the *Igκ* locus. **(A,B)** Feature enrichment **(A)** and transition matrix **(B)** for states identified by the EpiCSeg algorithm when three states are specified. **(C)** Proportion of the *Igκ* locus that is in each state. State 1 was labelled as the “Enhancer” state (E State), state 2 as the “Architectural” state (A state), and state 3 as the “Background” state (Bg state). **(D)** Percentage of regions in the A, E, and Bg state, over and surrounding all Vκ genes, active and inactive Vκ genes separately, or random regions of equivalent size distribution. Vκ genes have been scaled to the median gene length (525 bp), while distances surrounding the genes are not scaled. **(E)** Number of genes in each state, and the locations at which those states are present. **(F,G)** Enrichment relative to background of chromatin features characteristic of the E state over Vκ recombination signal sequences (RSSs) **(F)** and promoters **(G)** associated with each state. Fdr-adjusted *p*-values from a two-sided Wilcoxon rank sum test are shown for the difference in enrichment between A and E state-associated promoters or RSSs. All data are included for statistical testing, but to better visualise the data, some outliers are not displayed.

Despite the complexity of the locus, we observed that within-class homogeneity and between-class heterogeneity is maximised with just three states (Figures 5A,B). This number and the characteristic attributes of the states are strikingly similar to our previous analysis of the *Igh* locus (14). Accordingly, we labelled these states as follows: a “Background” (Bg) state, which comprises most of the locus and shows little enrichment for any chromatin features; an “Architectural” (A) state, in which CTCF and RAD21 are enriched; and an “Enhancer” (E) state, which is enriched for several TFs and histone modifications, including PU.1, PAX5, IRF4, MED1, IKAROS, and BRG1 (Figures 5A,C). We note that our choice of three states is subjective. Running the algorithm with a higher number of states results in segregation into smaller sub-states, which display a low enrichment for a subset of the features enriched for in the A or E states, and are frequently adjacent to similar states (shown for 4–8 states in Figure S4 in Supplementary Material). This suggests that they are not distinct from the A and E state, and that the three-state model is the most appropriate.

When we examined the distribution of these states over the Vκ genes, we found that the E state was highly enriched over both the gene promoters and RSSs, but depleted elsewhere (Figure 5D). The A state displayed only slight enrichment over the Vκ genes, but was more broadly enriched in a region approximately 1–3 kb upstream of the genes. These patterns of enrichment were particularly striking when only actively recombining Vκ genes were considered, whilst inactive genes were almost exclusively enriched in the Bg state. We identified a total of 92 Vκ genes that were associated with only the E state, at the promoter of 26 genes, at the RSS of 35 genes, and at both of these regions of 31 genes (Figure 5E). Only 11 genes were associated exclusively with the A state, while 10 genes were associated with both the A and the E state. This distribution of states is in contrast to the *Igh* locus, in which we observed association only with the V_H_ gene RSSs, and moreover, the two states were mutually exclusive, that is, no V_H_ genes were associated with both the A and the E state (14). Features associated with the E state at the *Igκ* locus, including PAX5, PU.1, IRF4, IKAROS, MED1 BRG1, E2A, and H3K4me2, were more enriched over E state compared to A state RSSs and promoters (Figures 5F,G). Median enrichment of CTCF and RAD21 was higher over A state promoters and RSSs, although these differences were not significant (Figure S5 in Supplementary Material).

**Figure 6:**
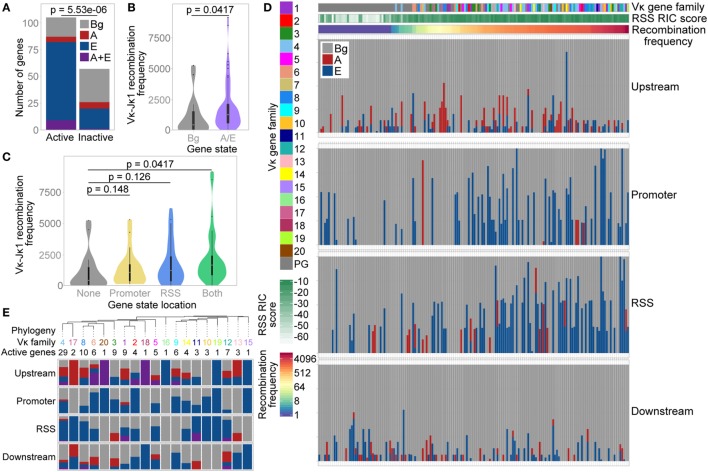
Vκ gene chromatin state is associated with recombination frequency. **(A)** Number of active and inactive genes in each state. *p-*value based on a Fisher’s exact test. **(B,C)** Violin plots with boxplot superimposed (black) showing median recombination frequencies of active genes in the Bg state compared to active genes associated with the A and/or E state. A and/or E state genes are considered altogether **(B)**, or categorised based on the localisation of the state to their promoter or recombination signal sequence (RSS) **(C)**. 10 genes that had <70% mappability were excluded. Fdr-adjusted *p*-values based on two-sided Wilcoxon rank sum test. **(D)** Proportion of each Vκ gene promoter and RSS (each window extending from the gene centre to 500 bp up/downstream of the gene, respectively; median window size 762 bp) and up- and downstream regions (from 500 to 3,500 bp up/downstream of the gene) that are assigned to each of the three states. Each bar represents an individual Vκ gene. Note that up- and downstream windows represent a genomic region approximately four times the size of the promoter and RSS windows. Genes are ordered based on their recombination frequency, with highly recombining genes on the right (denoted by red shading on the recombination frequency scale). Vκ gene family and RSS Information Content (RIC) score are indicated to the left and above. **(E)** Top: phylogenetic tree of reference C57BL/6 Vκ gene sequences, collapsed at the nodes containing the majority of each Vκ gene family. The number of active genes in each family is shown. All nodes comprise a single Vκ gene family except for one node, the Vκ9 family branch, which also includes Vκ14-130. Bottom: proportion of active genes in each family that are associated with the A state, E state, or with both states at their promoters, RSSs and up/downstream regions [colours as in **(A)**].

The distribution of these three states was significantly different over active versus inactive genes, with a much lower proportion of active genes in the Bg state. 83% of active V genes exhibited an E, A, or A/E chromatin state, compared with only 46% of inactive V genes (Figure 6A; Table S2 in Supplementary Material); the majority of these inactive genes had a RIC score below −25 (the lowest score for any active gene). Moreover, active genes marked by the A and/or E state recombine with significantly greater frequency than active genes in the Bg state (Figure 6B). When we compared the localisation of these states, the median recombination frequency of genes marked by the A and/or E state at either the promoter or the RSS was higher than that of Bg genes. The highest frequency was observed for genes marked in both regions, which was significantly different from Bg genes (Figure 6C). This is consistent with our analyses of individual ChIP-seq peaks above, suggesting that the presence of an active chromatin state at the RSS is more important in facilitating high levels of recombination than is an active state at the promoter. However, active chromatin states at both locations is particularly conducive to recombination.

Since we had observed an enrichment of the A state upstream of active Vκ genes, we also undertook a broader analysis of the states located close to each individual Vκ gene. As expected from our earlier analyses (Figures 6A–C), Vκ genes that recombine more frequently displayed a greater overlap with the E state in particular; this was apparent at both the promoter and the RSS (Figure 6D). Conversely, there was no clear relationship between recombination frequency and up- or downstream states, although this does not exclude a more complex influence of the surrounding chromatin on Vκ gene recombination.

We also compared the enrichment of chromatin states over a phylogenetic tree of Vκ gene families (Figure 6E). Considering only active genes, we observed some patterns in the state association of related families. For example, a large proportion of genes within the closely related Vκ4, Vκ17, Vκ8, and Vκ6 families (including in the two largest families, Vκ4 and Vκ8) are associated with the E state at their RSS; the A state was also frequently located upstream of these genes. However, unlike in the *Igh* locus (14), there was no clear evolutionary separation between genes associated with the A versus the E state. Indeed, the E state was also frequently associated with genes in the Vκ11, Vκ10, Vκ19, and Vκ12 families, which are closely related to each other but not to the families above.

We also asked whether the pre-recombination chromatin states at the Vκ gene promoters analysed here might in part explain the differences in the DNA Vκ-Jκ1 repertoire compared to the expressed repertoire (37), since these states may remain after recombination and additionally contribute to RNA expression. While 15 out of the top 20 most highly represented genes in the expressed repertoire were marked by either the A or E state at their promoters, 13 out of 20 Vκ genes that were present only in the DNA repertoire or that were highly represented in the DNA but had a low representation in the expressed repertoire, had promoters in the Bg state (Figure S6 in Supplementary Material). Thus, the chromatin state of the gene promoter can explain some of the differences in the repertoire. This underscores the value of using the DNA repertoire for these analyses, both to prevent the masking of the true recombination potential of each gene at the DNA level, and to ensure that conclusions drawn about the importance of chromatin features at the Vκ gene promoters prior to recombination are not confounded by differing expression levels.

### RSS RIC Score and PU.1 Binding Are Key Features That Distinguish Actively Recombining Genes from Inactive Genes

We next sought to understand how genetic and chromatin features regulate Vκ gene usage. Thus, we trained a RF-C to assess the power of each feature to correctly predict whether a gene is active or inactive; we have previously used this approach for analysis of *Igh* recombination (14). The RF-C takes features relating to each sample (here, each Vκ gene), and generates a large number of decision trees that vote on the response (here, Vκ gene recombination activity) for a training set of samples. At each step, one feature out of a random subset can be chosen, such that each tree will be unique. Feature importance is gauged by comparing the prediction accuracy for trees that include or exclude a given feature. The overall accuracy is then assessed by predicting the activity of an independent test set of genes. We chose the RF approach because it performs well both with a large number of features relative to the number of samples, and with highly correlated features (61).

**Figure 7:**
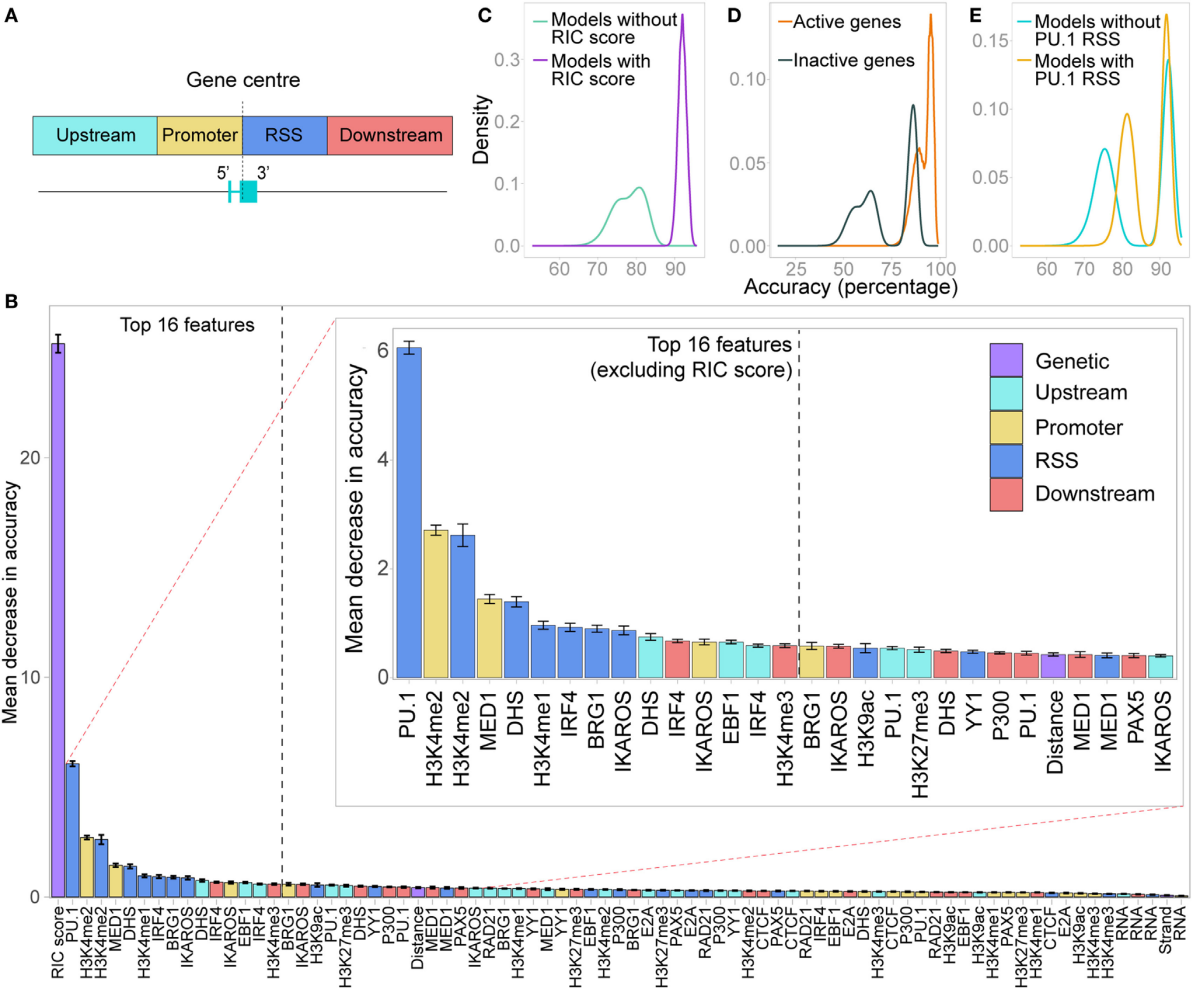
Random forest classifier (RF-C) distinguishes active from inactive genes. **(A)** Locations of the four windows in which chromatin features were quantified as an input to the random forest models. **(B)** Relative importance of all features in distinguishing active from inactive genes, shown as the average out-of-bag gini impurity (a measure of the decrease in accuracy if the feature is excluded) in a RF-C, with 10-fold cross-validation. Error bars show the SEM. Inset: zoomed-in view of the features ranked from 2 to 30 in importance. Features are coloured based on whether they are a genetic feature, or the location in which the chromatin feature was measured. **(C)** Overall prediction accuracy of all RF-C models trained with all combinations of the 16 most important features in the initial RF-C. Colour denotes whether the RSS Information Content (RIC) score was included in the model: all models that included the RIC score were more accurate than those that did not. **(D)** Accuracy in correctly predicting active genes versus inactive genes in all RF-C models. **(E)** Overall prediction accuracy for models that included PU.1 binding at the RSS as a feature, compared to those that did not.

We considered the signal intensity over the promoter separately from the signal intensity over the RSS, taking windows from the centre of each gene extending 500 bp upstream (promoter) or downstream (RSS) of the gene. We also calculated the signal intensities in windows extending a further 2.5 kb upstream of the promoter and downstream of the RSS (Figure 7A). In addition, we systematically gauged the contribution of the orientation of each Vκ gene and their genomic distance from *Jκ1* to facilitating recombination. Thus, the input for each gene included three genetic features: the RIC score, strand, and distance from *Jκ1*; and four separate features for each chromatin dataset (hereafter referred to as promoter, RSS, upstream and downstream).

A 10-fold cross-validation approach including all features revealed a mean prediction accuracy of 93.3% (SD 5.3%), with an F1 score of 0.949 (SD 0.0405). The prediction accuracy for active genes (97.2%, SD 6.2%) was better than that for inactive genes (85.7%, SD 14.5%), indicating a high sensitivity but slightly lower specificity in detecting active genes. The RIC score was by far the most important feature in distinguishing active from inactive genes (Figure 7B). The second most important feature was PU.1, which contributes to activation and recombination of the *Igκ* locus (21). Our model specifically suggests that while PU.1 binding at the promoter is of no consequence, its binding at the RSS is an important driver of recombination.

H3K4me2 enrichment within both the promoter and the RSS windows was also important for prediction accuracy, in addition to MED1 binding at the promoter and a number of E state-associated chromatin features at the RSS (Figure 7B). Notably, PAX5, CTCF, and RAD21, which are key drivers of *Igh* recombination (14), were not identified as important in promoting Vκ gene activity. Several up- or downstream features, such as DHS upstream and IRF4 binding downstream, were, however, ranked quite highly (Figure 7B), suggesting that in addition to the chromatin state of the Vκ genes themselves, the surrounding chromatin also influences the capacity of each gene to recombine.

Next, we performed a model selection analysis, considering all possible combinations of the 16 most important features. RF-C models that included the RIC score were all more accurate than those that did not (Figure 7C). This clear distinction was driven by the much lower prediction accuracy for inactive genes in RF-C models in which the RIC score was excluded (Figure 7D). There was also a striking contribution of PU.1 binding at the RSS to prediction accuracy, particularly in models that excluded the RIC score (Figure 7E); indeed, the bimodal distribution in prediction accuracy observed for models excluding the RIC score appears to be primarily dependent on this feature. This was apparent for both active and inactive genes (Figures S7A,B in Supplementary Material). We also observed a slight shift in prediction accuracy for models that included several other important features, such as IKAROS enrichment at the RSS (data not shown).

### Chromatin Features Alone Are Highly Predictive of the Recombination Frequency of Active Vκ Genes

**Figure 8:**
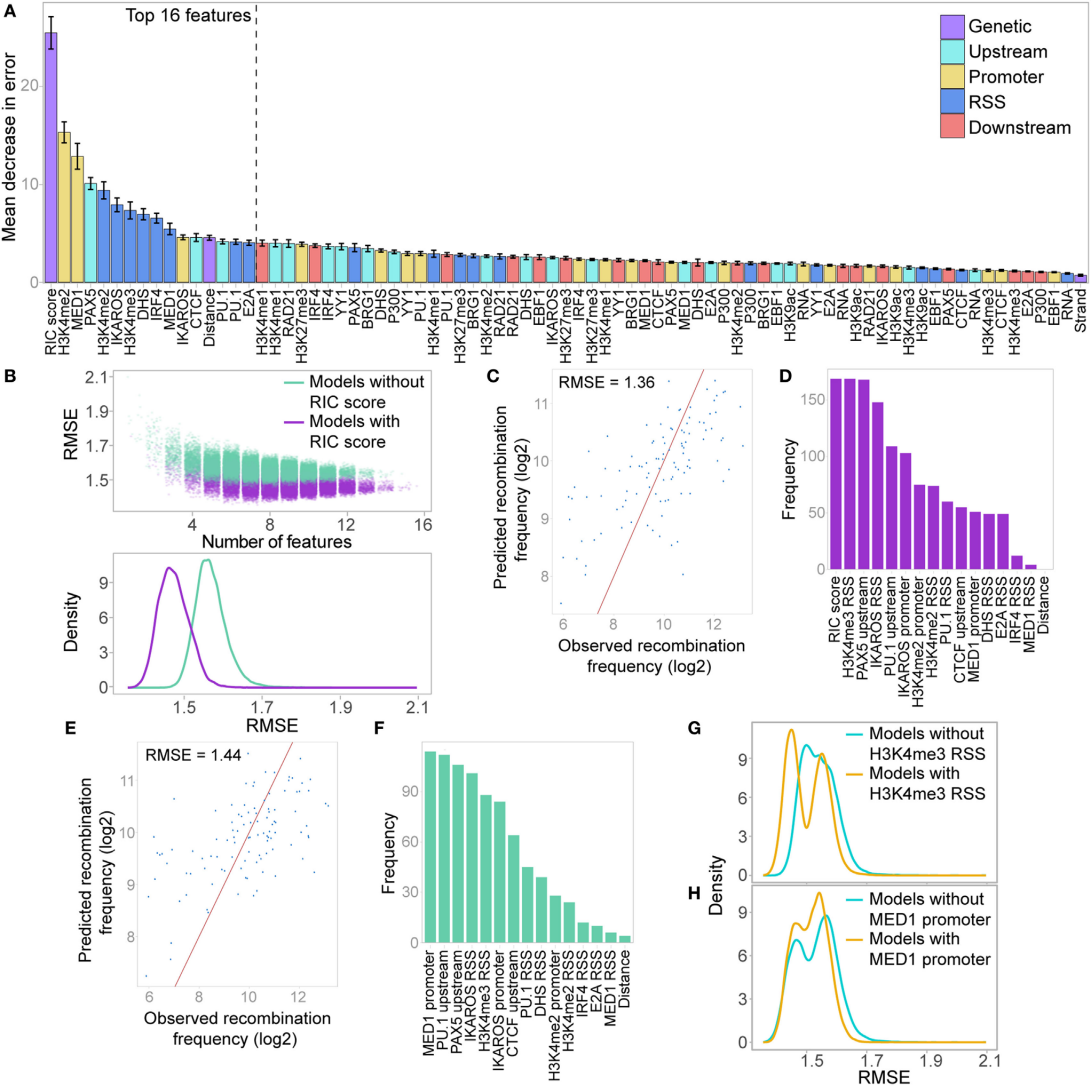
Random forest regression (RF-R) model for predicting recombination frequency of active Vκ genes. **(A)** Relative importance of all features in a RF-R model. Importance assessed by average out-of-bag node purity (a measure of the decrease in accuracy if the feature is excluded), with 10-fold cross validation. Error bars indicate the SEM. **(B)** Model selection, assessing all combinations of the 16 most important features from the initial RF-R model. Model performance is assessed as the root mean squared error (RMSE) across all test sets with 10-fold cross-validation. Colour denotes whether the RSS Information Content (RIC) score was included in the model as a feature. Top: scatter plot showing the RMSE of models with varying numbers of features; Bottom: density plot showing the distribution of the RMSE for all models that include or exclude the RIC score. **(C,E)** Observed versus predicted recombination frequencies across all test sets for the optimum RF-R model that included **(C)** or did not include **(E)** the RIC score as a feature, as assessed by RMSE. Observed = predicted is shown as a red line. **(D,F)** Frequency of inclusion of each feature in all models that included the RIC score and had an RMSE <1.39 **(D)**, or that excluded the RIC score and had an RMSE <1.48 **(F)**. **(G,H)** RMSE across all test sets for RF-R models that included H3K4me3 RSS **(G)** or MED1 promoter **(H)** as a feature compared to those that did not.

While the RF-C approach identifies the features that are most highly predictive of active Vκ gene recombination, and thus likely facilitate recombination, it does not directly show whether their levels contribute to the frequency with which an active gene is used. To address this, we trained a RF-R model for active Vκ genes, using the same set of features used for the classification model. This extends beyond the RF-C approach, giving a numerical prediction of the recombination frequency, with the log2-transformed recombination frequencies of active genes as the response variable. We used the RMSE to measure model performance. An initial RF-R using all features, with 10-fold cross-validation, achieved an RMSE of 1.57, indicating that 68% of the models’ predictions fall within 2^1.57^-fold (since recombination values are log2-transformed) of the observed recombination. This revealed the RIC score to be the most important feature for predicting the frequency of active Vκ gene recombination, as in the RF-C model (Figure 8A). However, there was far less separation between the importance of the RIC score and the other features. PU.1 binding at the RSS was much lower in importance compared to the RF-C model, while several features that were unimportant for classification contribute much more to the prediction of recombination frequency: these include PAX5 and CTCF binding upstream, and H3K4me3 and E2A binding at the RSS.

We next performed a model selection analysis, considering the top 16 features, to identify a minimum subset that best predict Vκ gene usage. While several combinations of 6–9 features have similarly low RMSE (Figure 8B), the minimum RMSE of 1.36 (Figure 8C) is achieved with a combination comprising: RIC score, H3K4me2, and H3K4me3 within the RSS window, IKAROS binding at both the promoter and the RSS, and PAX5 and PU.1 binding upstream. These seven features, in addition to H3K4me2 within the promoter window, were also the most highly represented in all models that had a low RMSE (<1.39), with the RIC score and H3K4me3 at the RSS being present in all models (Figure 8D). While this does not necessarily mean that these features are the most important individually (compare to Figure 8A), it suggests that together they are able to explain the largest proportion of the variability in our data. In models that excluded the RIC score, the best combination had an RMSE of 1.44 (Figure 8E), and comprised H3K4me3 at the RSS, IKAROS binding at both the promoter and the RSS, and PAX5 and PU.1 binding upstream, in addition to MED1 binding at the promoter. These six features, in addition to CTCF binding upstream, were the most frequently represented in models with low RMSE (<1.48) that excluded the RIC score, with MED1 binding to the promoter in all models (Figure 8F); this is particularly noteworthy since this feature was rarely present in the best combinations that included the RIC score. The inclusion of H3K4me3 at the RSS in a model was accompanied by a shift towards lower RMSE; this shift was more pronounced for the left-hand peak, corresponding to models that also include the RIC score (Figure 8G). Conversely, a shift towards lower RMSE for models that included MED1 binding at the promoter was only evident for the right-hand peak, representing models excluding the RIC score (Figure 8H). We also noted a shift towards a lower RMSE for models that included several other important features, including IKAROS binding at the promoter or RSS, or PAX5 or PU.1 binding upstream (Figure S7C in Supplementary Material).

These analyses revealed that we are able to predict the recombination frequency of Vκ genes from a combination of 6 or 7 features with a mean error rate of less than threefold, even when the RIC score is excluded (2.71-fold compared to 2.57-fold when including the RIC score). This model performance is highly significant when noting the variability of greater than 150-fold in recombination frequency across all active Vκ genes.

**Figure 9:**
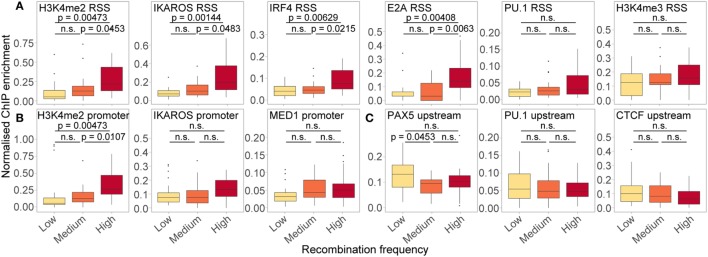
Relationship between ChIP enrichment and recombination frequency for important RSS **(A)**, promoter **(B)**, and upstream **(C)** features in RF models. Enrichment of chromatin features over the locations in which they were found to be important (projected between 0 and 1 for each gene: identical to the input for RF-R models), for active genes with low (*n* = 23; 117–973 reads), medium (*n* = 24; 1,006–1,801 reads) and high (*n* = 24; 1,880–9,137 reads) relative frequency of recombination. Only genes with a high quality RIC score (> −14) were considered. Fdr-adjusted *p*-values driven by two-sided Wilcoxon rank sum test. All data are included for statistical testing, but to better visualise the data, some outliers are not displayed.

In order to further dissect the influence of the chromatin features shown to be important in our RF models, we split all active genes with a good RIC score (>−14) into three groups based on their recombination frequency, and examined the enrichment of features of interest at the location(s) in which they were important (Figure 9). Genes that recombine more frequently tended to display higher levels of active histone modifications and TF binding at both the RSS (Figure 9A) and the promoter (Figure 9B). These trends were particularly striking for H3K4me2 (RSS and promoter windows) and IKAROS (RSS), in addition to IRF4 and E2A binding at the RSS, all of which displayed a significant positive relationship between recombination and enrichment. For PU.1 binding at the RSS, which was highly important in distinguishing active from inactive genes, the difference between genes that recombine at low and high frequency was more subtle (*p* > 0.05), consistent with the lower importance of this feature for predicting the frequency of recombination. We also noted a subtle, but non-significant, trend towards higher enrichment of MED1 and IKAROS at the promoter, and H3K4me3 at the RSS, of more highly recombining genes: the importance of these features in the RF-R thus suggests that a more complex relationship exists between these features and recombination frequency. Conversely, we noted a slight, negative association between the recombination frequency and the binding of some TFs upstream of the gene, including PAX5, PU.1, and CTCF, with a significantly greater enrichment of PAX5 upstream of genes that recombine at a low level compared to those that recombine at a medium level (Figure 9C).

## Discussion

We have adapted the VDJ-seq assay for the *Igκ* locus to quantitatively profile the Vκ-Jκ repertoire and to enable an in-depth analysis of the local drivers of recombination. Using cutting-edge random forest machine learning approaches to integrate genetic and chromatin features, we have distinguished genes that are actively recombining from those that are not, and have predicted the relative usage of active Vκ genes in primary recombination. We have found that local chromatin features, including PU.1 and IKAROS binding, and H3K4 methylation, explain much of the variation in recombination among Vκ genes.

The accuracy with which we can predict both Vκ gene activity and frequency of usage, even when the influence of the RIC score is excluded, is striking. Since we used pro-B cell genome-wide datasets, focussing on early events that prime the *Igκ* locus for recombination, the regulatory status of the *Igκ* locus may not fully reflect its state in pre-B cells immediately prior to Vκ-Jκ1 recombination. This suggests that early priming events are crucially important and that to a large extent, the recombination potential of each Vκ gene has been established by the pro-B cell stage. Nevertheless, we cannot exclude the possibility that features ranked unimportant here may become enriched at the *Igκ* locus later in development, or that additional pre-B cell specific features including IRF8, AIOLOS, and BRWD1 (27, 78, 79) may play a local regulatory role in recombination. Profiling of the locus in a *Rag^−/−^* model with a rearranged V_H_DJ_H_ transgene would reveal pre-B cell developmental activation signatures that might predict Vκ gene usage with even greater accuracy.

Our recent analysis of the *Igh* V_H_DJ_H_ repertoire (14) identified two mutually exclusive chromatin states, characterised by PAX5/IRF4 (enhancer/E state) or CTCF/RAD21 (architectural/A state) binding. Both localised exclusively to the RSSs of active V_H_ genes, and their characteristic features were highly predictive of active recombination. We found striking similarities in the regulation of the *Igκ* locus, but with several important differences. While the two chromatin states at the *Igκ* locus were similar to those at the *Igh* locus, the E state predominates at Vκ genes. Moreover, the states were associated with both promoters and RSSs of Vκ genes; indeed both regions were represented within the most important features identified by our RF models. The individual chromatin features that we identified as important in driving *Igκ* recombination (e.g., PU.1) were also substantially different from those driving *Igh* recombination. Furthermore, our RF-R model, which was not used to assess *Igh* recombination, allowed us to take this analysis to the next level, giving a numerical prediction of recombination frequency. This approach allowed us to distinguish chromatin features that play a binary, all-or-nothing role in Vκ recombination from those that fine-tune the repertoire, shaping the frequency with which active genes will recombine.

Our RF-C model identified the RIC score as the most important feature for distinguishing actively recombining genes from inactive genes. Nevertheless, we achieved greater than 80% prediction accuracy based purely on chromatin features. This was primarily dependent on PU.1 binding at the RSS. PU.1 binding to the *Igκ* 3′ enhancer has been implicated in activation and recombination of the *Igκ* locus (23). In addition, Batista and colleagues observed frequent binding of PU.1 at Vκ RSSs and hypothesised that this may play a role in recruiting RAG enzymes for recombination (21). Our analysis provides direct mechanistic insight, revealing that PU.1 binding at the RSS of Vκ genes is a critical binary switch, which dictates whether that Vκ gene will recombine or not. Interestingly, PU.1 was not important in *Igh* V_H_DJ_H_ recombination (14); conversely, CTCF, RAD21 and PAX5 binding, which were critical for *Igh* recombination, were unimportant in our *Igκ* RF-C model. We found IRF4 and DHS to be significant for recombination at both Ig loci. While H3K4 methylation featured strongly in both models, monomethylation was more prominent for V_H_ genes in the *Igh* locus, while dimethylation was more important in shaping the VκJκ1 repertoire. Thus, the local regulation of *Igh* and *Igκ* by histone modifications and TFs differs considerably. Features associated with the A state in particular appear to play a less significant role in priming the Vκ genes for recombination.

The RIC score was also the most important feature in our RF-R model. However, the distinction between it and the most important chromatin features was much lower than for the RF-C model, suggesting that similar to the *Igh* locus, the RIC score functions primarily as a binary switch that can be permissive or non-permissive to recombination.

Surprisingly, the influence of PU.1 binding to the RSS also appears to be binary: while it was key to the RF-C model, its importance in the RF-R model was much lower. Rather, the features with the greatest importance in predicting the frequency of recombination in the RF-R model included IKAROS, MED1, IRF4, and H3K4 di- and tri-methylation at the promoter and/or RSS. Moreover, at more frequently recombining genes, significant trends towards higher levels of enrichment were observed for H3K4me2, IKAROS, IRF4, and E2A. This suggests that both the binding and level of enrichment of these features are crucial for modulating the frequency with which each gene recombines, shaping the greater than 150-fold variation in active Vκ gene usage in the primary repertoire. While each of these have previously been implicated in promoting *Igκ* locus recombination (18, 22, 24, 25, 27, 39, 40, 75), our findings provide mechanistic insight into their specific roles in shaping the repertoire through their localisation to individual Vκ genes. First, their locations at the promoter/RSS provide an additional layer of regulation beyond previously observed long-range interactions. Second, the correlation of higher levels of enrichment with higher recombination, measured here in bulk populations, suggests that these features localise to the relevant Vκ genes in a higher proportion of individual cells, or remain associated with these Vκ genes for longer, with a functional outcome of increased recombination.

A caveat of the RF approach is that it does not directly show how a given feature is related to Vκ gene recombination; the finite number of genes also means that over-fitting of the data could be a concern, although the use of 10-fold cross-validation mitigates this possibility. Nevertheless, the clear relationship that we observed between enrichment and recombination for several features, including IKAROS and IRF4, highlights the value of this approach in providing a shortlist of chromatin features that are potential drivers of recombination. The contribution of other features that did not display such a clear relationship with recombination, such as H3K4me3 at the RSS and MED1 binding at the promoter, will require further work to elucidate their roles. H3K4me3 binds and activates RAG2 (17–20), suggesting a direct role in recruitment of the RAG complex. However, to our knowledge, this is the first time that MED1 has been implicated in recombination of the *Igκ* locus.

Notably, we did not observe a significant contribution of non-coding transcription to either RF model, suggesting that transcription does not play a predictive role in Vκ recombination. A previous study proposed that transcription causes the eviction of H2A/H2B around Vκ RSSs (75), and non-coding transcription has also been shown to mark recombinationally active domains of the Vκ region (16). Together, these findings suggest that, in common with histone H3 and H4 acetylation, non-coding transcription may play a priming role for all Vκ genes, setting the stage for the features we have described here to specifically activate Vκ genes for recombination with a range of frequencies.

We also identified the binding of PAX5, CTCF, and PU.1 upstream of Vκ genes as relatively important in predicting the recombination frequency of active genes, and observed subtle negative relationships between recombination frequency and enrichment of these features. While determining the mechanisms that underpin these relationships are beyond the scope of this study, it is noteworthy that PAX5 and CTCF have been implicated in long-range looping of the *Igh* and *Igh*/*Igκ* loci, respectively (30, 32–35, 40, 80), bringing V genes into proximity with the (D)J genes. Thus, it is tempting to speculate that the intergenic sites bound by these TFs might correspond to the anchors of these loops. Looping of the locus promotes the recombination of distal genes; however, genes located very close to loop anchors might be spatially constrained, disfavouring their recombination. Conversely, H3K4 methylation, E2A, and IKAROS binding have also been implicated in looping of the *Igκ* locus, but previous data, consistent with our RF models, suggest a positive correlation with recombination (39, 40). Notably, while CTCF is generally located between the Vκ genes, the other features were localised to the genes themselves. Furthermore, CTCF-associated interactions were confined to the SIS regulatory element, a silencer located between the Vκ and Jκ genes (81, 82), while loci marked by the other features also interacted with the *Igκ* enhancers. This suggests different classes of loops might exist, with CTCF and PAX5 required for the overall global architecture of the locus, bringing genes into the vicinity of the Jκ region. This might allow other TFs, such as E2A and IKAROS, to mediate the local clustering of active genes immediately adjacent to the Jκ genes and the recruitment of the *Igκ* enhancers to these genes. More detailed analysis of the three dimensional structure of the *Igκ* locus, and its relationship with the chromatin features implicated in its organisation, will be required to establish the validity of this hypothesis.

In addition to V(D)J recombination at the AgR loci, our findings also have implications for RAG-mediated off-target recombination events throughout the genome, which can lead to leukaemias. Promoter and enhancer signatures are associated with high frequency, genome-wide recruitment of the RAG complex (42–44). Our previous study allowed us to refine those signatures by identifying that features of the A and E state are enriched at these sites (14). Here, we have identified additional candidates (PU.1, IKAROS) that may enhance predictive models of chromosomal translocation hotspots (43).

The findings reported here demonstrate that the mechanisms that regulate Vκ recombination differ substantially from those that regulate V_H_ recombination. They also identify two distinct and crucial roles for chromatin features in regulating Vκ gene recombination. While PU.1 binding at the RSS plays a binary role in priming Vκ genes to recombine, the binding and variable enrichment of several other chromatin features, including H3K4 methylation, IKAROS binding at the RSS, and MED1 binding at the promoter, modulate the frequency with which each active gene recombines. Furthermore, inclusion of this canonical signature may refine prediction of genome-wide RAG1 binding sites susceptible to chromosomal translocation.

## Ethics Statement

C57BL/6 (WT) and Rag1^−/−^/VH81X mice were maintained in accordance with Babraham Institute AWERB and Home Office rules and ARRIVE guidelines under Project Licence 80/2529.

## Author Contributions

LM developed the VκJκ-seq assay reported here and generated VκJκ-seq data. FK and SA adapted the Babraham LinkON pipeline for the Igκ locus. LM, DB, and PC refined the VκJκ-seq protocol and analysis pipeline. HK pre-processed and Q.C. checked all NGS datasets. LM and HK performed computational and machine learning analyses. LM visualised the data and prepared the figures. LM, HK, and AC interpreted the results. LM and AC wrote the manuscript.

## Conflict of Interest Statement

LM, DB, and AC are named inventors on a patent filed, “Covering the VDJ-seq technique: method of identifying VDJ recombination products.” (UK Patent Application No. GB1203720.6, filed March 2, 2012; PCT Patent Applic No. PCT/GB2013/05056, published September 6, 2013. National applications filed Europe, USA, Japan. US Publication number: 20150031042, publication date January 29, 2015.). All other authors declare that the research was conducted in the absence of any commercial or financial relationships that could be construed as a potential conflict of interest.

## Footnotes

^1^https://github.com/FelixKrueger/BabrahamLinkON.

^2^http://www.bioinformatics.babraham.ac.uk/projects/trim_galore/.

^3^http://www.bioinformatics.babraham.ac.uk/projects/seqmonk/.

^4^https://www.ncbi.nlm.nih.gov/nuccore/NG_005612/.

^5^https://www.ncbi.nlm.nih.gov/geo/query/acc.cgi?acc=GSE101606.

## Abbreviations

AgR, antigen receptor; BCR, B cell receptor; D, Diversity; DHS, DNase hypersensitivity; *Igh*, Immunoglobulin heavy chain; *Igκ*, Immunoglobulin kappa light chain; J, joining; RF-C, random forest classification; RF-R, random forest regression; RSS, recombination signal sequence; RMSE, root mean squared error; RIC, RSS information content; TdT, terminal deoxynucleotidyl transferase; TF, transcription factor; V, variable; WT, wild type.

## References

[1] Fugmann SD Lee AI Shockett PE Villey IJ Schatz DG. The RAG proteins and V(D)J recombination: complexes, ends, and transposition. Annu Rev Immunol (2000) 18:495–527. 10.1146/annurev.immunol.18.1.495 10837067

[2] Jung D Giallourakis C Mostoslavsky R Alt FW. Mechanism and control of V(D)J recombination at the immunoglobulin heavy chain locus. Annu Rev Immunol (2006) 24:541–70. 10.1146/annurev.immunol.23.021704.115830 16551259

[3] Benedict CL Gilfillan S Thai TH Kearney JF. Terminal deoxynucleotidyl transferase and repertoire development. Immunol Rev (2000) 175:150–7. 10.1111/j.1600-065X.2000.imr017518.x 10933600

[4] Hendriks RW Middendorp S The pre-BCR checkpoint as a cell-autonomous proliferation switch. Trends Immunol (2004) 25(5):249–56. 10.1016/j.it.2004.02.011 15099565

[5] Herzog S Reth M Jumaa H. Regulation of B-cell proliferation and differentiation by pre-B-cell receptor signalling. Nat Rev Immunol (2009) 9(3):195–205. 10.1038/nri2491 19240758

[6] Brekke KM Garrard WT. Assembly and analysis of the mouse immunoglobulin kappa gene sequence. Immunogenetics (2004) 56(7):490–505. 10.1007/s00251-004-0659-0 15378297

[7] Li YS Hayakawa K Hardy RR. The regulated expression of B lineage associated genes during B cell differentiation in bone marrow and fetal liver. J Exp Med (1993) 178(3):951–60. 10.1084/jem.178.3.951 8350062 PMC2191150

[8] Victor KD Vu K Feeney AJ. Limited junctional diversity in kappa light chains. Junctional sequences from CD43+B220+ early B cell progenitors resemble those from peripheral B cells. J Immunol (1994) 152(7):3467–75. 7511648

[9] Bertocci B De Smet A Berek C Weill JC Reynaud CA. Immunoglobulin kappa light chain gene rearrangement is impaired in mice deficient for DNA polymerase mu. Immunity (2003) 19(2):203–11. 10.1016/S1074-7613(03)00203-6 12932354

[10] Nemazee D. Receptor editing in lymphocyte development and central tolerance. Nat Rev Immunol (2006) 6(10):728–40. 10.1038/nri1939 16998507

[11] Vettermann C Timblin GA Lim V Lai EC Schlissel MS. The proximal J kappa germline-transcript promoter facilitates receptor editing through control of ordered recombination. PLoS One (2015) 10(1):e0113824. 10.1371/journal.pone.0113824 25559567 PMC4283955

[12] Cowell LG Davila M Kepler TB Kelsoe G. Identification and utilization of arbitrary correlations in models of recombination signal sequences. Genome Biol (2002) 3(12):RESEARCH0072. 10.1186/gb-2002-3-12-research0072 12537561 PMC151174

[13] Lee AI Fugmann SD Cowell LG Ptaszek LM Kelsoe G Schatz DG. A functional analysis of the spacer of V(D)J recombination signal sequences. PLoS Biol (2003) 1(1):E1. 10.1371/journal.pbio.0000001 14551903 PMC212687

[14] Bolland DJ Koohy H Wood AL Matheson LS Krueger F Stubbington MJ Two mutually exclusive local chromatin states drive efficient V(D)J recombination. Cell Rep (2016) 15(11):2475–87. 10.1016/j.celrep.2016.05.020 27264181 PMC4914699

[15] Choi NM Loguercio S Verma-Gaur J Degner SC Torkamani A Su AI Deep sequencing of the murine IgH repertoire reveals complex regulation of nonrandom V gene rearrangement frequencies. J Immunol (2013) 191(5):2393–402. 10.4049/jimmunol.1301279 23898036 PMC3778908

[16] Levin-Klein R Fraenkel S Lichtenstein M Matheson LS Bartok O Nevo Y Clonally stable Vkappa allelic choice instructs Igkappa repertoire. Nat Commun (2017) 8:15575 10.1038/ncomms15575 28555639 PMC5459994

[17] Bettridge J Na CH Pandey A Desiderio S. H3K4me3 induces allosteric conformational changes in the DNA-binding and catalytic regions of the V(D)J recombinase. Proc Natl Acad Sci U S A (2017) 114(8):1904–9. 10.1073/pnas.1615727114 28174273 PMC5338453

[18] Liu Y Subrahmanyam R Chakraborty T Sen R Desiderio S. A plant homeodomain in RAG-2 that binds hypermethylated lysine 4 of histone H3 is necessary for efficient antigen-receptor-gene rearrangement. Immunity (2007) 27(4):561–71. 10.1016/j.immuni.2007.09.005 17936034 PMC3711682

[19] Matthews AG Kuo AJ Ramon-Maiques S Han S Champagne KS Ivanov D RAG2 PHD finger couples histone H3 lysine 4 trimethylation with V(D)J recombination. Nature (2007) 450(7172):1106–10. 10.1038/nature06431 18033247 PMC2988437

[20] Shimazaki N Tsai AG Lieber MR. H3K4me3 stimulates the V(D)J RAG complex for both nicking and hairpinning in trans in addition to tethering in cis: implications for translocations. Mol Cell (2009) 34(5):535–44. 10.1016/j.molcel.2009.05.011 19524534 PMC2920491

[21] Batista CR Li SK Xu LS Solomon LA DeKoter RP. PU.1 regulates Ig light chain transcription and rearrangement in pre-B cells during B cell development. J Immunol (2017) 198(4):1565–74. 10.4049/jimmunol.1601709 28062693

[22] Heizmann B Kastner P Chan S. Ikaros is absolutely required for pre-B cell differentiation by attenuating IL-7 signals. J Exp Med (2013) 210(13):2823–32. 10.1084/jem.20131735 24297995 PMC3865471

[23] Hodawadekar S Park K Farrar MA Atchison ML A developmentally controlled competitive STAT5-PU.1 DNA binding mechanism regulates activity of the Ig kappa E3’ enhancer. J Immunol (2012) 188(5):2276–84. 10.4049/jimmunol.1102239 22279106 PMC3288515

[24] Inlay MA Tian H Lin T Xu Y. Important roles for E protein binding sites within the immunoglobulin kappa chain intronic enhancer in activating Vkappa Jkappa rearrangement. J Exp Med (2004) 200(9):1205–11. 10.1084/jem.20041135 15504821 PMC2211861

[25] Johnson K Hashimshony T Sawai CM Pongubala JM Skok JA Aifantis I Regulation of immunoglobulin light-chain recombination by the transcription factor IRF-4 and the attenuation of interleukin-7 signaling. Immunity (2008) 28(3):335–45. 10.1016/j.immuni.2007.12.019 18280186

[26] Lazorchak AS Schlissel MS Zhuang Y. E2A and IRF-4/Pip promote chromatin modification and transcription of the immunoglobulin kappa locus in pre-B cells. Mol Cell Biol (2006) 26(3):810–21. 10.1128/MCB.26.3.810-821.2006 16428437 PMC1347029

[27] Ma S Turetsky A Trinh L Lu R. IFN regulatory factor 4 and 8 promote Ig light chain kappa locus activation in pre-B cell development. J Immunol (2006) 177(11):7898–904. 10.4049/jimmunol.177.11.7898 17114461

[28] Sakamoto S Wakae K Anzai Y Murai K Tamaki N Miyazaki M E2A and CBP/p300 act in synergy to promote chromatin accessibility of the immunoglobulin kappa locus. J Immunol (2012) 188(11):5547–60. 10.4049/jimmunol.1002346 22544934

[29] Sato H Saito-Ohara F Inazawa J Kudo A. Pax-5 is essential for kappa sterile transcription during Ig kappa chain gene rearrangement. J Immunol (2004) 172(8):4858–65. 10.4049/jimmunol.172.8.4858 15067064

[30] Degner SC Verma-Gaur J Wong TP Bossen C Iverson GM Torkamani A CCCTC-binding factor (CTCF) and cohesin influence the genomic architecture of the Igh locus and antisense transcription in pro-B cells. Proc Natl Acad Sci U S A (2011) 108(23):9566–71. 10.1073/pnas.1019391108 21606361 PMC3111298

[31] Guo C Yoon HS Franklin A Jain S Ebert A Cheng HL CTCF-binding elements mediate control of V(D)J recombination. Nature (2011) 477(7365):424–30. 10.1038/nature10495 21909113 PMC3342812

[32] Ribeiro de Almeida C Stadhouders R de Bruijn MJ Bergen IM Thongjuea S Lenhard B The DNA-binding protein CTCF limits proximal Vkappa recombination and restricts kappa enhancer interactions to the immunoglobulin kappa light chain locus. Immunity (2011) 35(4):501–13. 10.1016/j.immuni.2011.07.014 22035845

[33] Xiang Y Park SK Garrard WT Vkappa gene repertoire and locus contraction are specified by critical DNase I hypersensitive sites within the Vkappa-Jkappa intervening region. J Immunol (2013) 190(4):1819–26. 10.4049/jimmunol.1203127 23296705 PMC3563863

[34] Xiang Y Zhou X Hewitt SL Skok JA Garrard WT A multifunctional element in the mouse Igkappa locus that specifies repertoire and Ig loci subnuclear location. J Immunol (2011) 186(9):5356–66. 10.4049/jimmunol.1003794 21441452 PMC3080443

[35] Fuxa M Skok J Souabni A Salvagiotto G Roldan E Busslinger M. Pax5 induces V-to-DJ rearrangements and locus contraction of the immunoglobulin heavy-chain gene. Genes Dev (2004) 18(4):411–22. 10.1101/gad.291504 15004008 PMC359395

[36] Liu H Schmidt-Supprian M Shi Y Hobeika E Barteneva N Jumaa H Yin Yang 1 is a critical regulator of B-cell development. Genes Dev (2007) 21(10):1179–89. 10.1101/gad.1529307 17504937 PMC1865490

[37] Aoki-Ota M Torkamani A Ota T Schork N Nemazee D Skewed primary Igkappa repertoire and V-J joining in C57BL/6 mice: implications for recombination accessibility and receptor editing. J Immunol (2012) 188(5):2305–15. 10.4049/jimmunol.1103484 22287713 PMC3288532

[38] Lin SG Ba Z Du Z Zhang Y Hu J Alt FW. Highly sensitive and unbiased approach for elucidating antibody repertoires. Proc Natl Acad Sci U S A (2016) 113(28):7846–51. 10.1073/pnas.1608649113 27354528 PMC4948367

[39] Lin YC Benner C Mansson R Heinz S Miyazaki K Miyazaki M Global changes in the nuclear positioning of genes and intra- and interdomain genomic interactions that orchestrate B cell fate. Nat Immunol (2012) 13(12):1196–204. 10.1038/ni.2432 23064439 PMC3501570

[40] Stadhouders R de Bruijn MJ Rother MB Yuvaraj S Ribeiro de Almeida C Kolovos P Pre-B cell receptor signaling induces immunoglobulin kappa locus accessibility by functional redistribution of enhancer-mediated chromatin interactions. PLoS Biol (2014) 12(2):e1001791 10.1371/journal.pbio.1001791 24558349 PMC3928034

[41] Pan X Papasani M Hao Y Calamito M Wei F Quinn WJ III YY1 controls Igkappa repertoire and B-cell development, and localizes with condensin on the Igkappa locus. EMBO J (2013) 32(8):1168–82. 10.1038/emboj.2013.66 23531880 PMC3630362

[42] Ji Y Resch W Corbett E Yamane A Casellas R Schatz DG. The in vivo pattern of binding of RAG1 and RAG2 to antigen receptor loci. Cell (2010) 141(3):419–31. 10.1016/j.cell.2010.03.010 20398922 PMC2879619

[43] Maman Y Teng G Seth R Kleinstein SH Schatz DG. RAG1 targeting in the genome is dominated by chromatin interactions mediated by the non-core regions of RAG1 and RAG2. Nucleic Acids Res (2016) 44(20):9624–37. 10.1093/nar/gkw633 27436288 PMC5175335

[44] Teng G Maman Y Resch W Kim M Yamane A Qian J RAG represents a widespread threat to the lymphocyte genome. Cell (2015) 162(4):751–65. 10.1016/j.cell.2015.07.009 26234156 PMC4537821

[45] Feddersen RM Van Ness BG. Corrective recombination of mouse immunoglobulin kappa alleles in Abelson murine leukemia virus-transformed pre-B cells. Mol Cell Biol (1990) 10(2):569–76. 10.1128/MCB.10.2.569 2153918 PMC360841

[46] Yamagami T ten Boekel E Andersson J Rolink A Melchers F. Frequencies of multiple IgL chain gene rearrangements in single normal or kappaL chain-deficient B lineage cells. Immunity (1999) 11(3):317–27. 10.1016/S1074-7613(00)80107-7 10514010

[47] Martin F Chen X Kearney JF. Development of VH81X transgene-bearing B cells in fetus and adult: sites for expansion and deletion in conventional and CD5/B1 cells. Int Immunol (1997) 9(4):493–505. 10.1093/intimm/9.4.493 9138009

[48] Mombaerts P Iacomini J Johnson RS Herrup K Tonegawa S Papaioannou VE. RAG-1-deficient mice have no mature B and T lymphocytes. Cell (1992) 68(5):869–77. 10.1016/0092-8674(92)90030-G 1547488

[49] Langmead B Trapnell C Pop M Salzberg SL. Ultrafast and memory-efficient alignment of short DNA sequences to the human genome. Genome Biol (2009) 10(3):R25. 10.1186/gb-2009-10-3-r25 19261174 PMC2690996

[50] Alamyar E Duroux P Lefranc MP Giudicelli V IMGT((R)) tools for the nucleotide analysis of immunoglobulin (IG) and T cell receptor (TR) V-(D)-J repertoires, polymorphisms, and IG mutations: IMGT/V-QUEST and IMGT/HighV-QUEST for NGS. Methods Mol Biol (2012) 882:569–604. 10.1007/978-1-61779-842-9_32 22665256

[51] Zhang Y Liu T Meyer CA Eeckhoute J Johnson DS Bernstein BE Model-based analysis of ChIP-Seq (MACS). Genome Biol (2008) 9(9):R137. 10.1186/gb-2008-9-9-r137 18798982 PMC2592715

[52] Dereeper A Audic S Claverie JM Blanc G. BLAST-EXPLORER helps you building datasets for phylogenetic analysis. BMC Evol Biol (2010) 10:8. 10.1186/1471-2148-10-8 20067610 PMC2821324

[53] Dereeper A Guignon V Blanc G Audic S Buffet S Chevenet F Phylogeny.fr: robust phylogenetic analysis for the non-specialist. Nucleic Acids Res (2008) 36(Web Server issue):W465–9. 10.1093/nar/gkn180 18424797 PMC2447785

[54] Edgar RC. MUSCLE: multiple sequence alignment with high accuracy and high throughput. Nucleic Acids Res (2004) 32(5):1792–7. 10.1093/nar/gkh340 15034147 PMC390337

[55] Anisimova M Gascuel O. Approximate likelihood-ratio test for branches: a fast, accurate, and powerful alternative. Syst Biol (2006) 55(4):539–52. 10.1080/10635150600755453 16785212

[56] Guindon S Gascuel O. A simple, fast, and accurate algorithm to estimate large phylogenies by maximum likelihood. Syst Biol (2003) 52(5):696–704. 10.1080/10635150390235520 14530136

[57] Yu G Smith DK Zhu H Guan Y Lam TT-Y McInerny G ggtree: an R package for visualization and annotation of phylogenetic trees with their covariates and other associated data. Methods Ecol Evol (2017) 8(1):28–36. 10.1111/2041-210x.12628

[58] Mammana A Chung HR. Chromatin segmentation based on a probabilistic model for read counts explains a large portion of the epigenome. Genome Biol (2015) 16:151. 10.1186/s13059-015-0708-z 26206277 PMC4514447

[59] Ernst J Kellis M ChromHMM: automating chromatin-state discovery and characterization. Nat Methods (2012) 9(3):215–6. 10.1038/nmeth.1906 22373907 PMC3577932

[60] Quinlan AR Hall IM. BEDTools: a flexible suite of utilities for comparing genomic features. Bioinformatics (2010) 26(6):841–2. 10.1093/bioinformatics/btq033 20110278 PMC2832824

[61] Boulesteix AL Janitza S Kruppa J Konig IR Overview of random forest methodology and practical guidance with emphasis on computational biology and bioinformatics. Wiley Interdiscip Rev Data Min Knowl Discov (2012) 2(6):493–507. 10.1002/widm.1072

[62] Liaw A Wiener M Classification and regression by randomForest. R News (2002) 2/3:18–22.

[63] Langmead B Salzberg SL. Fast gapped-read alignment with Bowtie 2. Nat Methods (2012) 9(4):357–9. 10.1038/nmeth.1923 22388286 PMC3322381

[64] Revilla-i-Domingo R Bilic I Vilagos B Tagoh H Ebert A Tamir IM The B-cell identity factor Pax5 regulates distinct transcriptional programmes in early and late B lymphopoiesis. EMBO J (2012) 31(14):3130–46. 10.1038/emboj.2012.155 22669466 PMC3400013

[65] Ebert A McManus S Tagoh H Medvedovic J Salvagiotto G Novatchkova M The distal V(H) gene cluster of the Igh locus contains distinct regulatory elements with Pax5 transcription factor-dependent activity in pro-B cells. Immunity (2011) 34(2):175–87. 10.1016/j.immuni.2011.02.005 21349430

[66] Medvedovic J Ebert A Tagoh H Tamir IM Schwickert TA Novatchkova M Flexible long-range loops in the VH gene region of the Igh locus facilitate the generation of a diverse antibody repertoire. Immunity (2013) 39(2):229–44. 10.1016/j.immuni.2013.08.011 23973221 PMC4810778

[67] Mullen AC Orlando DA Newman JJ Loven J Kumar RM Bilodeau S Master transcription factors determine cell-type-specific responses to TGF-beta signaling. Cell (2011) 147(3):565–76. 10.1016/j.cell.2011.08.050 22036565 PMC3212730

[68] Whyte WA Orlando DA Hnisz D Abraham BJ Lin CY Kagey MH Master transcription factors and mediator establish super-enhancers at key cell identity genes. Cell (2013) 153(2):307–19. 10.1016/j.cell.2013.03.035 23582322 PMC3653129

[69] Vilagos B Hoffmann M Souabni A Sun Q Werner B Medvedovic J Essential role of EBF1 in the generation and function of distinct mature B cell types. J Exp Med (2012) 209(4):775–92. 10.1084/jem.20112422 22473956 PMC3328360

[70] Schwickert TA Tagoh H Gultekin S Dakic A Axelsson E Minnich M Stage-specific control of early B cell development by the transcription factor Ikaros. Nat Immunol (2014) 15(3):283–93. 10.1038/ni.2828 24509509 PMC5790181

[71] Lin YC Jhunjhunwala S Benner C Heinz S Welinder E Mansson R A global network of transcription factors, involving E2A, EBF1 and Foxo1, that orchestrates B cell fate. Nat Immunol (2010) 11(7):635–43. 10.1038/ni.1891 20543837 PMC2896911

[72] Bossen C Murre CS Chang AN Mansson R Rodewald HR Murre C. The chromatin remodeler Brg1 activates enhancer repertoires to establish B cell identity and modulate cell growth. Nat Immunol (2015) 16(7):775–84. 10.1038/ni.3170 25985234 PMC4474778

[73] Spanopoulou E Roman CA Corcoran LM Schlissel MS Silver DP Nemazee D Functional immunoglobulin transgenes guide ordered B-cell differentiation in Rag-1-deficient mice. Genes Dev (1994) 8(9):1030–42. 10.1101/gad.8.9.1030 7926785

[74] Predeus AV Gopalakrishnan S Huang Y Tang J Feeney AJ Oltz EM Targeted chromatin profiling reveals novel enhancers in Ig H and Ig L chain loci. J Immunol (2014) 192(3):1064–70. 10.4049/jimmunol.1302800 24353267 PMC4096788

[75] Bevington S Boyes J. Transcription-coupled eviction of histones H2A/H2B governs V(D)J recombination. EMBO J (2013) 32(10):1381–92. 10.1038/emboj.2013.42 23463099 PMC3655464

[76] Goldmit M Ji Y Skok J Roldan E Jung S Cedar H Epigenetic ontogeny of the Igk locus during B cell development. Nat Immunol (2005) 6(2):198–203. 10.1038/ni1154 15619624

[77] Kleiman E Jia H Loguercio S Su AI Feeney AJ. YY1 plays an essential role at all stages of B-cell differentiation. Proc Natl Acad Sci U S A (2016) 113(27):E3911–20. 10.1073/pnas.1606297113 27335461 PMC4941496

[78] Ma S Pathak S Trinh L Lu R. Interferon regulatory factors 4 and 8 induce the expression of Ikaros and Aiolos to down-regulate pre-B-cell receptor and promote cell-cycle withdrawal in pre-B-cell development. Blood (2008) 111(3):1396–403. 10.1182/blood-2007-08-110106 17971486 PMC2214771

[79] Mandal M Hamel KM Maienschein-Cline M Tanaka A Teng G Tuteja JH Histone reader BRWD1 targets and restricts recombination to the Igk locus. Nat Immunol (2015) 16(10):1094–103. 10.1038/ni.3249 26301565 PMC4575638

[80] Guo C Alt FW Giallourakis C. PAIRing for distal Igh locus V(D)J recombination. Immunity (2011) 34(2):139–41. 10.1016/j.immuni.2011.02.010 21349424

[81] Liu Z Widlak P Zou Y Xiao F Oh M Li S A recombination silencer that specifies heterochromatin positioning and ikaros association in the immunoglobulin kappa locus. Immunity (2006) 24(4):405–15. 10.1016/j.immuni.2006.02.001 16618599

[82] Liu ZM George-Raizen JB Li S Meyers KC Chang MY Garrard WT. Chromatin structural analyses of the mouse Igkappa gene locus reveal new hypersensitive sites specifying a transcriptional silencer and enhancer. J Biol Chem (2002) 277(36):32640–9. 10.1074/jbc.M204065200 12080064

