# Supplementary material for: Local Chromatin Features Including PU.1 and IKAROS Binding and H3K4 Methylation Shape the Repertoire of Immunoglobulin Kappa Genes Chosen for V(D)J Recombination
Source: Front Immunol. 2017 Nov 17;8:1550. doi: 10.3389/fimmu.2017.01550 (PMC5698286; doi:10.3389/fimmu.2017.01550)
Supplement: Supplementary file 2 [file Data_Sheet_1.PDF]

Supplementary Figures and Text

Local chromatin features including PU.1 and IKAROS binding and H3K4 methylation shape the repertoire of immunoglobulin kappa genes chosen for V(D)J recombination

Louise S. Matheson, Daniel J. Bolland, Peter Chovanec, Felix Krueger, Simon Andrews, Hashem Koohy*, Anne E. Corcoran*

***Correspondence:** Anne Corcoran: anne.corcoran@babraham.ac.uk

Hashem Koohy: hashem.koohy@rdm.ox.ac.uk

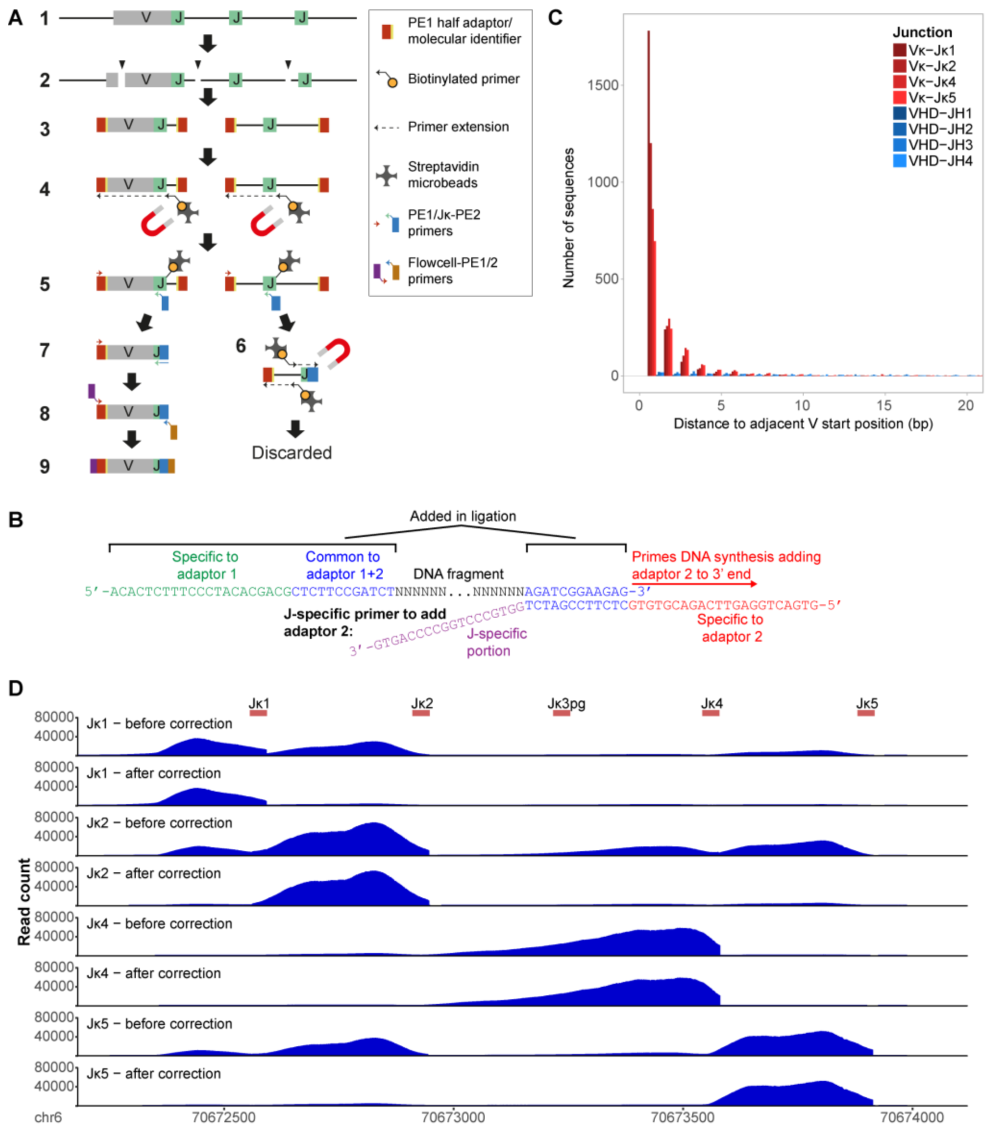

Supplementary Figure S1. Adaptation and optimisation of the VDJ-seq assay for the Igκ locus. **(A)** Flowchart showing the optimised VκJκ-seq assay. 1. Vκ-Jκ recombined DNA. 2. Sonication to 400 bp (arrowheads) using Covaris E220. 3. End repair, A-tailing and ligation of adaptor 1 and molecular identifier. 4. Primer extension with biotinylated primers annealing downstream of each Jκ gene (dashed line) and enrichment of products using streptavidin-coated magnetic beads. 5. Limited enrichment of Jκ-containing products by PCR with PE1 and Jκ-PE2 primers. 6. Second primer extension with biotinylated primers annealing upstream of each Jκ gene and removal of unrecombined products. 7. Further enrichment of Jκ-containing products by PCR. 8. Addition of flowcell-binding portions of PE1 and PE2 adaptors by PCR. 9. Libraries ready for Illumina sequencing (2 x 100 bp). **(B)** Without carbon spacer, reverse adaptor added to the 3' end of all DNA fragments during ligation of adaptor 1 can be used as a primer in the later J-specific PCR reaction, allowing the addition of adaptor 2 to random DNA fragments and thus introducing background. **(C)** Total number of V reads across the five most common V(D)J junctions for each JH and Jκ gene, plotted based on their separation from the closest downstream V read associated with the same junction sequence. **(D)** Read counts across the Jκ region when the J gene is assigned before or after mis-priming correction. These reads originate from the incomplete depletion of germline sequences, and would be expected to map upstream of the J gene to which they are assigned.

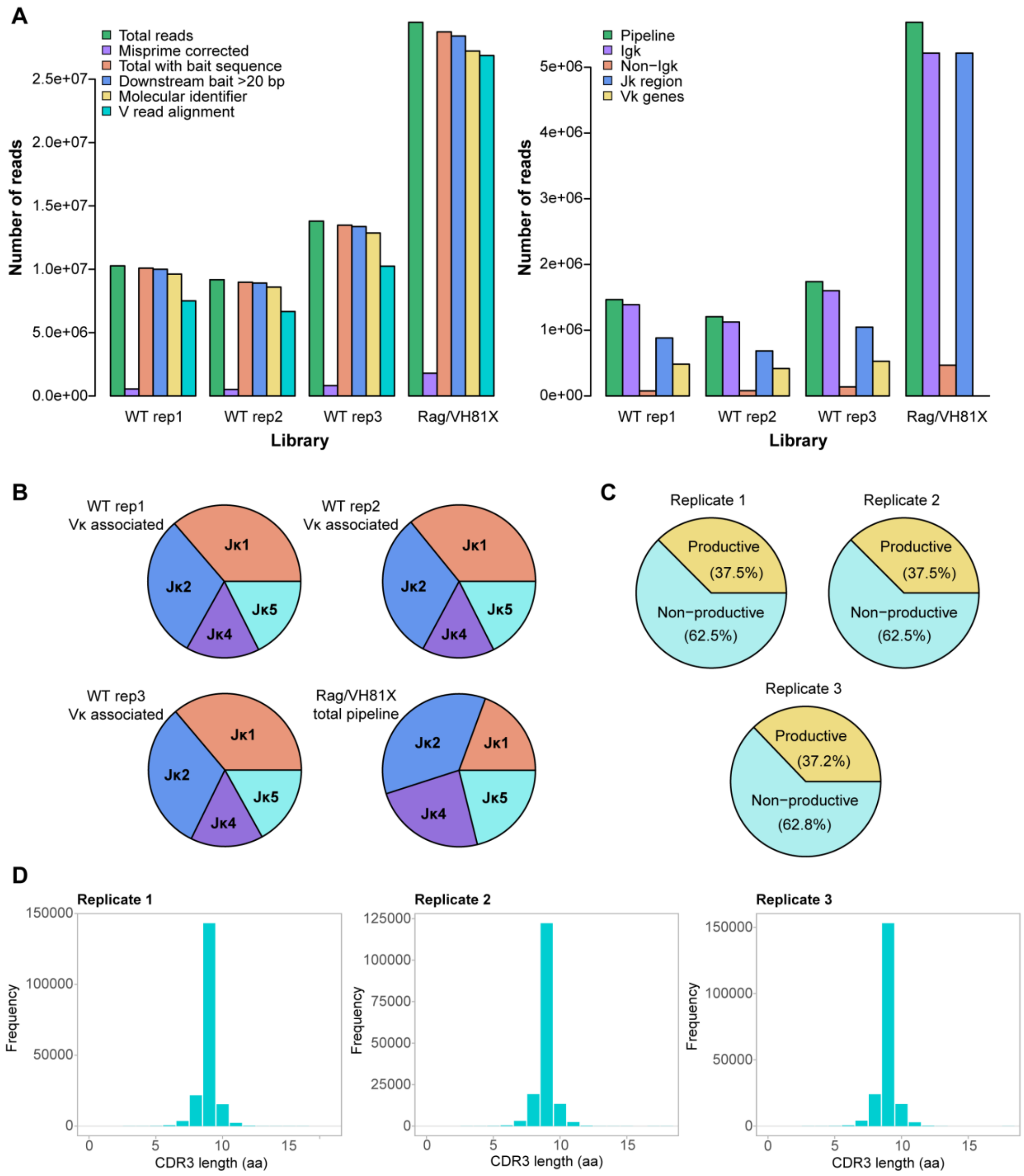

Supplementary Figure S2. Quality control for VκJκ-seq libraries. **(A)** General statistics for each library. Left – total numbers of reads for each library passing each stage of the analysis pipeline, including the number of sequences corrected for mis-priming. Right – total number of V-end alignments remaining after deduplication, and the locations to which they map. **(B)** Proportion of reads associated with each Jκ gene, after deduplication, including all alignments (Rag/VH81X) or only Vκ alignments (WT replicates 1-3). **(C, D)** IMGT-HighVQUEST analysis of productivity **(C)** and CDR3 length **(D)** for merged V and J reads from each replicate.

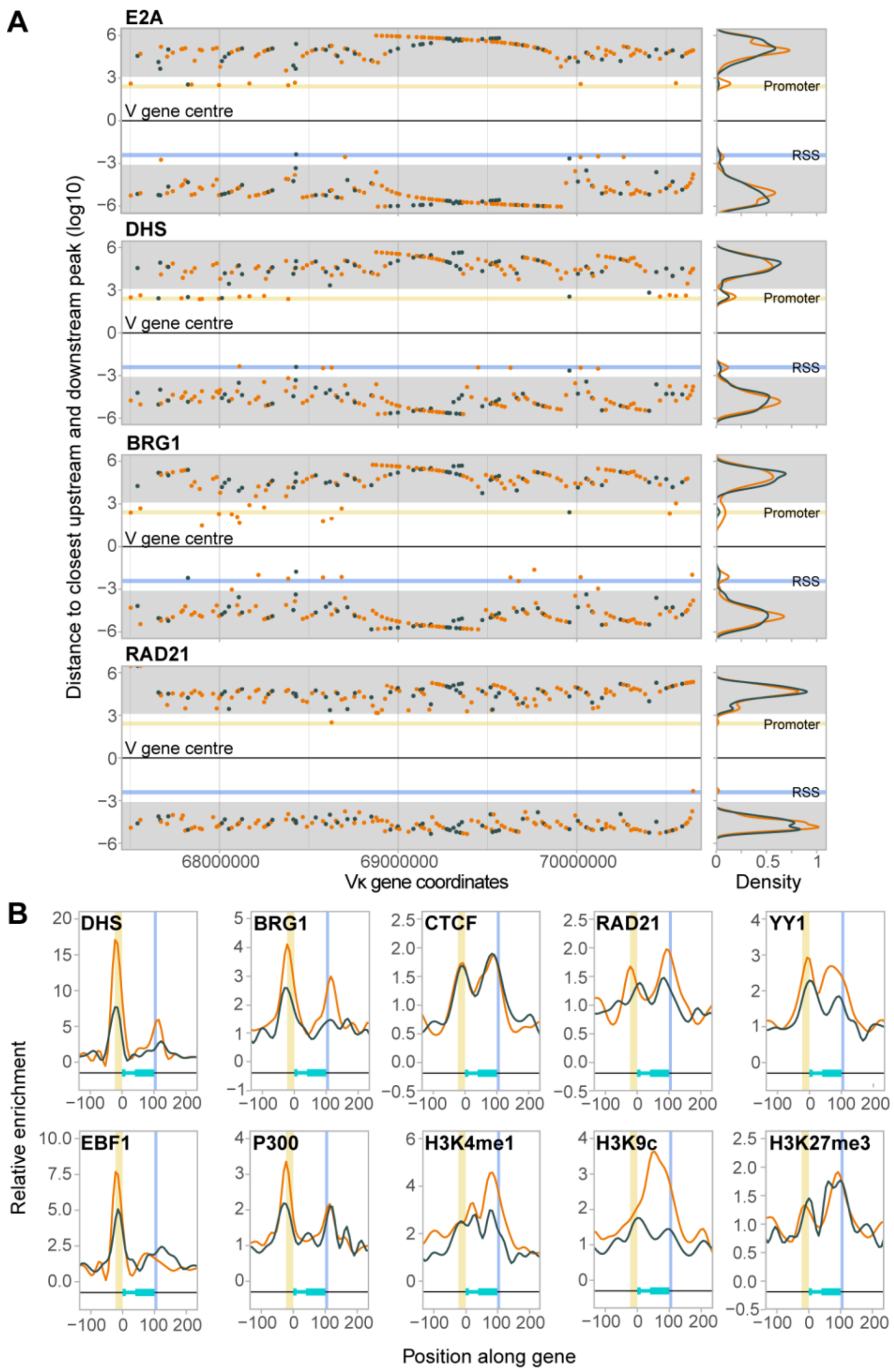

Supplementary Figure S3. Chromatin features associate with both the promoters and RSSs of active Vκ genes. **(A)** Scatter plots and density plots showing the log10-transformed distances of the closest ChIP-seq peaks both up- (+) and downstream (-) from the centre of active (orange) and inactive (dark grey) Vκ genes. Yellow (promoter) and blue (RSS) shading indicates the range of distances within which 80% of the start and end sites of Vκ genes, respectively, are located. Grey shading indicates a distance of > 1 kb from the Vκ gene, based on the median Vκ gene length (525 bp). **(B)** Average enrichment, relative to background, of chromatin features across all active (orange) and inactive (dark grey) Vκ genes. Genes have been scaled such that 0 and 100 represent the start and end of the gene respectively. Yellow and blue shading indicates the location of the promoter and RSS respectively.

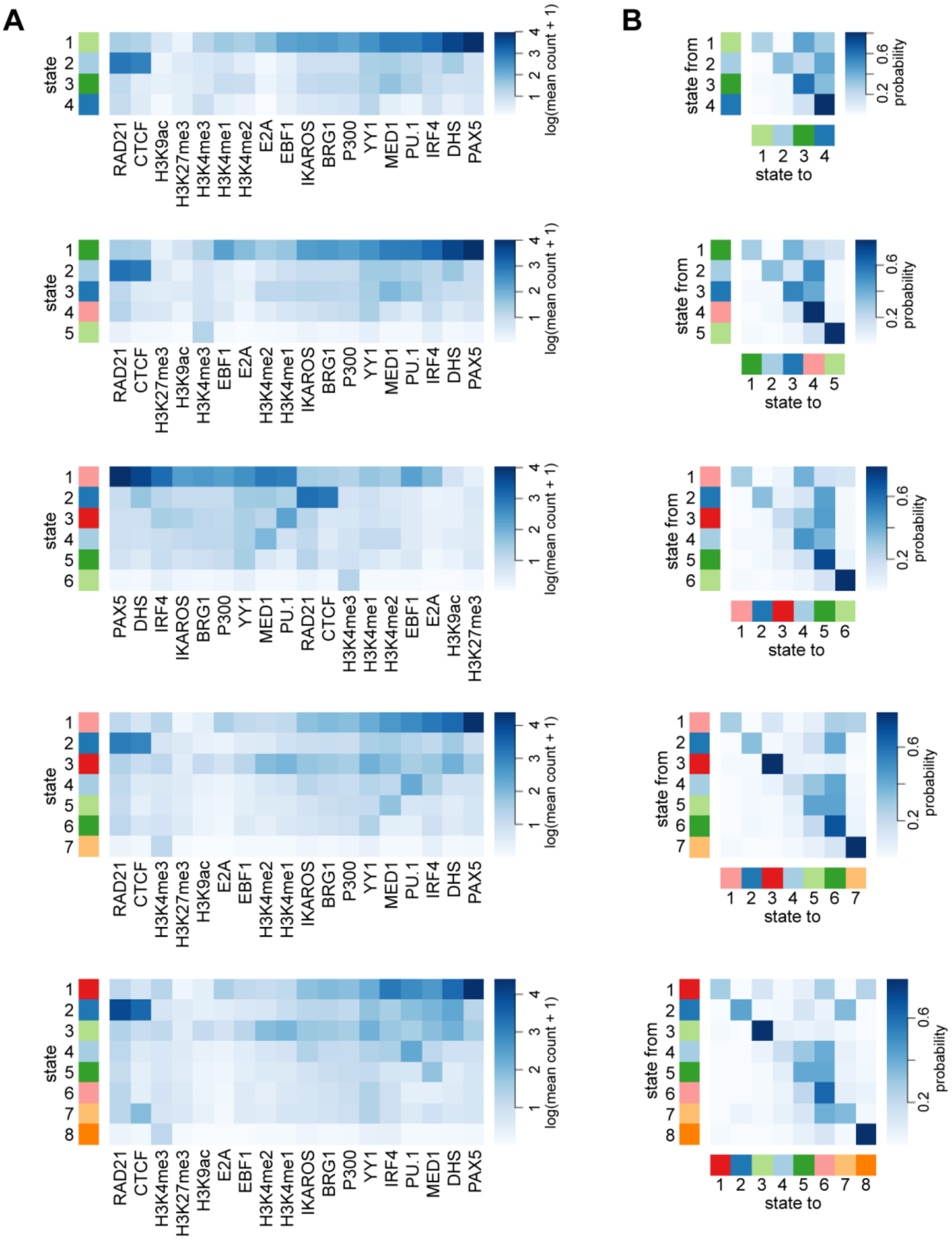

Supplementary Figure S4. Comparison of chromatin segmentation upon specifying varying numbers of states. **(A, B)** Feature enrichment **(A)** and transition matrices **(B)** for states identified by the EpiCSeg algorithm when 4-8 states are specified. In comparison to the 3-state model (Figure 5A, B), when the number of states is increased, the additional states are comprised of subsets of the original three states **(A)**, with frequent transitions between similar states **(B)**, suggesting that they are not truly distinct.

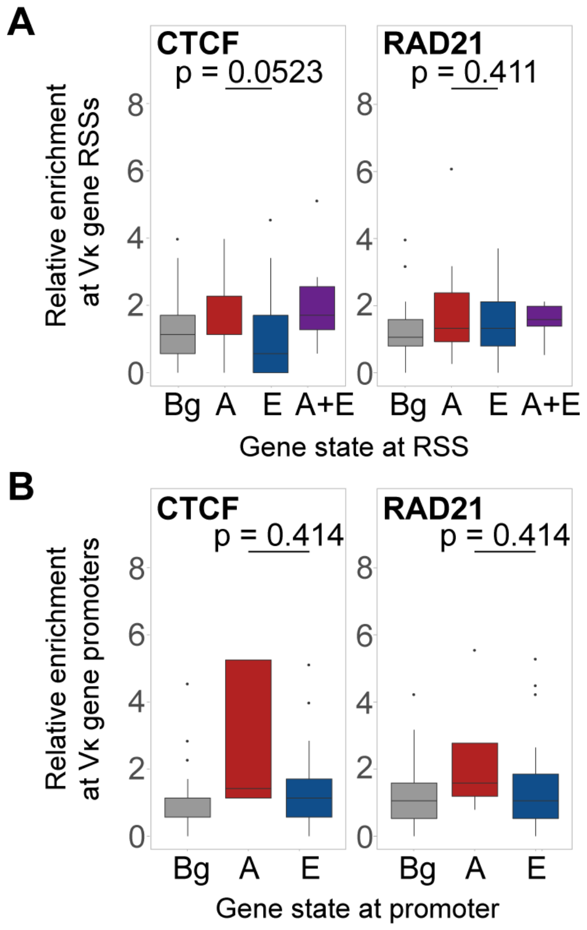

Supplementary Figure S5. Enrichment relative to background of chromatin features characteristic of the A state over Vκ RSSs (F) and promoters (G) associated with each state. Fdr-adjusted p values from a two-sided Wilcoxon rank sum test are shown for the difference in enrichment between A and E state-associated promoters or RSSs. All data is included for statistical testing, but to better visualise the data, some outliers are not displayed.

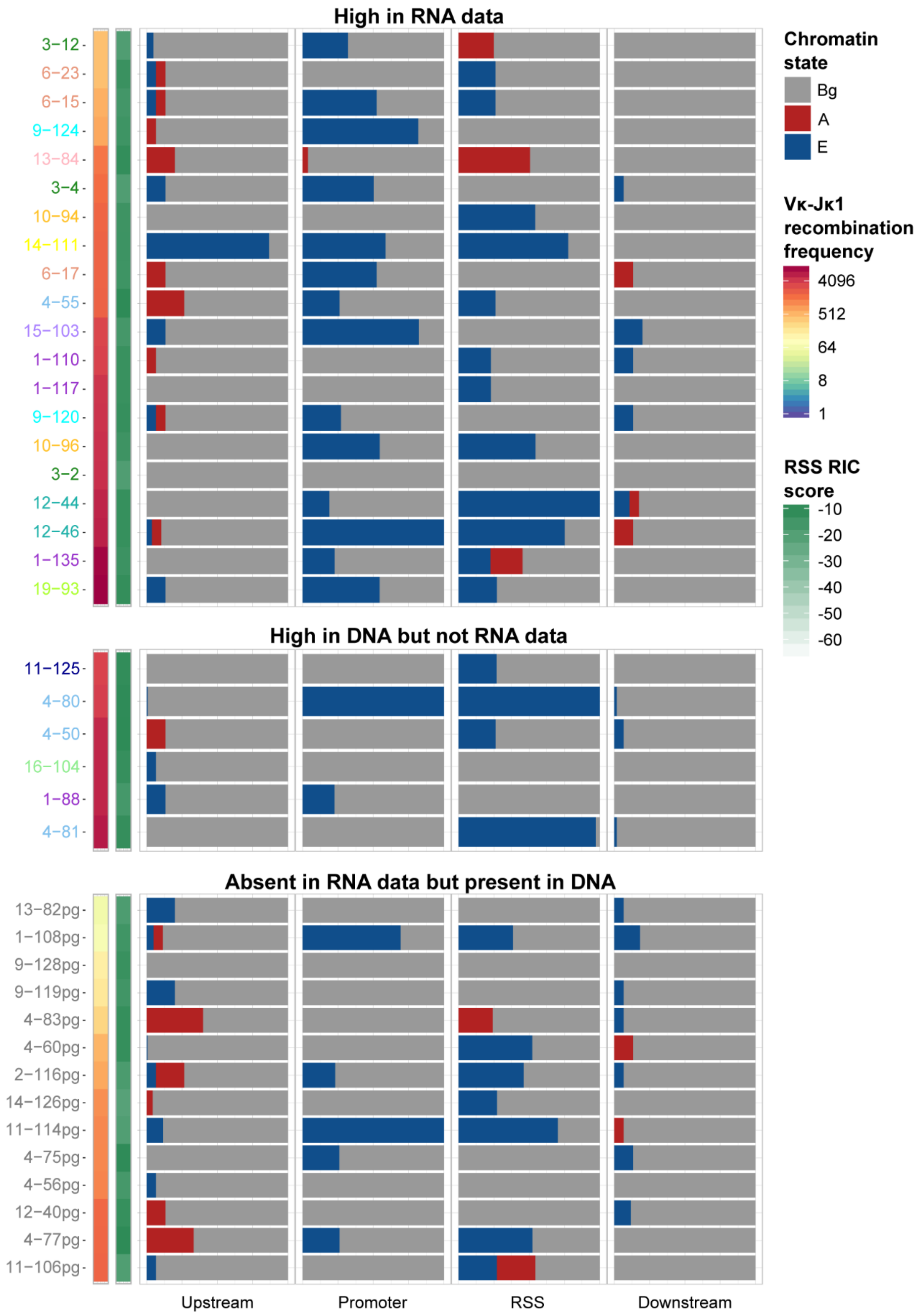

Supplementary Figure S6. Chromatin states of genes with high or low representation in the RNA repertoire. Proportion of each window around selected Vκ genes that are present at high or low frequency in the RNA repertoire (37). Each bar represents a single Vκ gene, indicated on the left, with the colour of the label denoting the Vκ gene family; pseudogenes (pg) are displayed in grey. Top: The top 20 genes in the RNA repertoire. Middle: Genes that are high in the DNA repertoire but low in the RNA repertoire. Bottom: Genes that are absent from the RNA repertoire but actively recombined in the DNA repertoire.

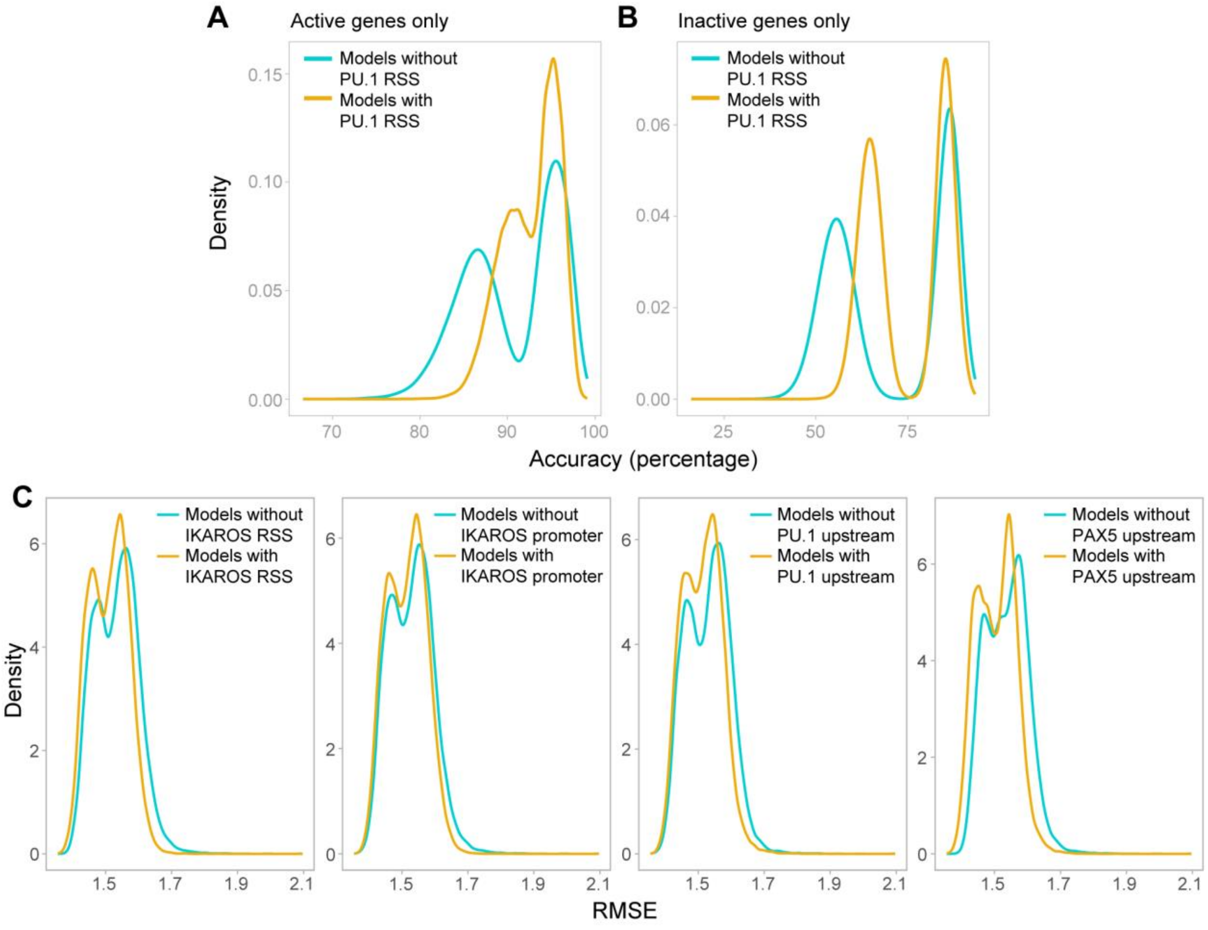

Supplementary Figure S7. Model selection for RF-C and RF-R. **(A, B)** Accuracy in predicting active **(A)** or inactive **(B)** genes in RF-C models that included PU.1 binding at the RSS compared to those that did not. **(C)** RMSE of RF-R models that included the indicated features that were identified as important, compared to those that did not

Supplementary Text S1. Optimisation and adaptation of the VDJ-seq assay for the Igκ locus

Adaptations to the VDJ-seq assay

In addition to designing primers against the four functional Jκ genes (Jκ1, 2, 4 and 5; Supplementary Table S4), modifications were made to improve the assay and to make it suitable for the Igκ locus.

First, for the initial shearing, genomic DNA was sonicated to 400 bp rather than 500 bp, since the Jκ genes are closer together than are the J_H genes, and VκJκ junctions are shorter than V_HDJ_H junctions. Second, a carbon spacer was added to the end of the reverse adapter, preventing it from being used as a primer in subsequent reactions (Supplementary Figure S1B) and thus reducing background. Third, the depletion step was performed after the enrichment and a limited number of PCR cycles. This was to ensure that VκJκ rearrangements that are on DNA fragments also containing the depletion target sequence for the downstream Jκ gene are not lost from the assay, and resulted in an increased enrichment in reads originating from recombined VκJκ genes (data not shown). Finally, the VκJκ rearrangements are much less diverse than V_HDJ_H rearrangements, firstly because they involve only two gene segments, and secondly because recombination is less imprecise due both to the lack of TdT expression, and thus vastly reduced incorporation of non-templated nucleotides (7, 8), and to the prevention of extensive nibbling of coding ends (9). In the Igh assay, unique recombination events were readily distinguished based on the sequence of the junction and the start location of the V_H/D_H read, which results from random DNA shearing. To determine whether this was also sufficient for the Igκ assay, we identified the most commonly occurring junction sequences for each Jκ gene, and mapped the Vκ reads for each after deduplication based on the V read start position, as detailed below. This revealed greater than ten-fold more reads for commonly occurring Vκ-Jκ junctions compared to V_HD-J_H junctions (Supplementary Figure S1C). Moreover, for the Vκ-Jκ junctions the vast majority of V start positions were separated by only 1 bp from the V start position of the adjacent read, indicating that the assay was saturated and that it is not possible to distinguish identical rearrangements from PCR duplicates purely based on the junction sequence and the Vκ mapping position. We therefore incorporated six random nucleotides into the adapters ligated onto the Vκ end of each fragment, which will be sequenced at the start of the Vκ reads, acting as molecular identifiers. These random nucleotides were followed by an anchor sequence of 6 or 7 bp preceding the T overhang to ensure efficient ligation; the use of two different anchor sequences was required to give sufficient diversity during sequencing.

The analysis pipeline was adjusted to take into account the molecular identifiers in calling unique recombination events. Analysis of the Jκ reads for unrecombined fragments indicated that some mis-priming of Jκ genes was occurring, similar to the J2-J4 mis-priming observed for the Igh assay (14). We identified the commonly occurring chimeric Jκ reads within the unrecombined fragments, indicative of mis-priming, and corrected these through replacement of the primer sequence prior to calling the Jκ gene (Supplementary Figure S1D).

Analysis of common junctions

For a preliminary VκJκ-seq library, and one of our published Igh VDJ-seq libraries ((14); sample GSM2113574), we first excluded read 2 sequences whose 3' end mapped within the J or D (for Igh) regions, and then identified the 10 most commonly occurring read 2 sequences following the bait for each J gene; these sequences include the V(D)J junctions. We then

mapped the V ends (read 1) for each junction, and deduplicated based on the mapping position, generating a separate BAM file for each junction. From these, we examined the V alignments, to identify the 5 most common V(D)J rearrangements that had identical junctions, for each J gene. For each of these, we then calculated the distance from the start of each V alignment to the start of the closest V alignment in the 3’ direction (with respect to the V gene) that was associated with the same junction. The distances for the 5 junctions analysed for a given J gene were pooled. If there are many V alignments for a given junction that all map very close to each other, this is an indication that the assay is saturated, since it is highly likely that some sequences with identical junctions and V mapping positions originated from different rearrangements, rather than as a result of PCR duplication.

Supplementary Text S2. Correction of Vκ gene annotations

When examining the reads aligned to the Vκ region, we noted some discrepancies when comparing to the gene annotations (based on NCBI Reference Sequence: NG_005612.1; available at https://www.ncbi.nlm.nih.gov/nuccore/NG_005612/). First, Vκ8-23-1 is annotated as being in the forward orientation, whereas all of the reads aligning to this gene were in the reverse orientation. A strong RSS is located at the annotated start of the gene; moreover, alignment of the sequence to other Vκ8 family members indicated that the gene is, indeed, in the reverse and not the forward orientation. Second, the reads aligning to Vκ4-60pg extended to cover approximately 100 bp downstream of the annotated gene end; we noted the presence of a strong RSS (RIC score -11.28) located 94 bp beyond the end of the gene. Alignment of the sequence to other Vκ4 family genes confirmed that this RSS marks the true 3' end of the gene. For both of these genes, we corrected the annotations before performing any further analyses.
